# Understanding cerebral autoregulation and cerebrovascular reactivity: mechanistic perspectives and clinical insights from TBI

**DOI:** 10.1186/s13054-026-06207-2

**Published:** 2026-08-04

**Authors:** Ihsane Olakorede, Stefan Yu Bögli, Marek Czosnyka, Peter Smielewski

**Affiliations:** 1 Brain Physics Laboratory, Division of Neurosurgery, Department of Clinical Neurosciences, University of Cambridge, Cambridge, UK; 2 Department of Neurology and Neurocritical Care Unit, Clinical Neuroscience Center, University Hospital Zurich, University of Zurich, Zurich, Switzerland; 3 Institute of Electronic Systems, Warsaw University of Technology, Warsaw, Poland

**Keywords:** Brain homeostasis, Cerebral autoregulation, Cerebral blood flow, Intracranial pressure, Neuromonitoring

## Abstract

Cerebrovascular reactivity and cerebral blood flow autoregulation are critical determinants of brain homeostasis but are often conflated in clinical practice. Although these constructs are closely related and arise from vascular responses to vasoactive stimuli, they reflect distinct physiological responses and measurement approaches: autoregulation is assessed through changes in cerebral blood flow, and pressure reactivity through changes in cerebral blood volume. A fundamental clinical challenge is that the full autoregulatory curve is rarely observable in an individual patient at a single point in time; consequently, isolated measurements from classical assessments or continuous indices such as the pressure reactivity index (PRx), mean flow index (Mx), and arterial mean flow index (Mxa) are each associated with different components of cerebrovascular physiology, providing only partial views of cerebrovascular function and frequently yielding apparently conflicting results. This Review addresses a central clinical question: can multimodal neuromonitoring help determine where a patient is operating on the autoregulatory curve? We synthesise current understanding of the physiological relationships among cerebral perfusion pressure, cerebral blood flow, and cerebral blood volume, and examine the mechanisms underlying divergent autoregulatory index behaviour. By integrating mechanistic physiological schematics and representative clinical cases, we propose an objective educational framework that links classical cerebrovascular physiology with contemporary bedside monitoring. Within this framework, six conceptual physiological zones are delineated. These zones demonstrate how variations in vascular tone, intracranial compliance, and critical closing pressure shape autoregulatory responses generate differing patterns in PRx, Mx, and Mxa. Fully defining a patient’s complex cerebrovascular state requires looking beyond any single parameter. Together, PRx, Mx, and Mxa provide complementary information, where discordant findings may represent distinct physiological mechanisms rather than measurement error. Interpreting these indices together within a mechanistic framework may improve identification of a patient’s position along the autoregulatory curve, support more consistent bedside interpretation, and ultimately facilitate personalised cerebral perfusion pressure management.

**Supplementary Information:**

The online version contains supplementary material available at https://doi.org/10.1186/s13054-026-06207-2.

## Introduction

Secondary brain injury remains a major determinant of outcome following traumatic brain injury (TBI). Optimising cerebral perfusion is therefore a cornerstone of modern neurocritical care, yet bedside assessment of cerebrovascular function remains challenging because cerebral blood flow (CBF) is governed by multiple interacting physiological mechanisms responding to changes in cerebral perfusion pressure (CPP), metabolic demand, vasoactive compounds, and neuronal activity.

Understanding these processes is particularly important in TBI, where impaired cerebrovascular regulation may contribute to secondary brain insults and worse clinical outcomes [1–7]. As a result, continuous assessment of cerebral autoregulation has emerged as a promising strategy for individualising haemodynamic management, by tailoring CPP to each patient’s physiological state rather than relying on universal treatment thresholds [8], although its clinical implementation remains challenging. In this setting, clinicians rarely observe the full autoregulatory curve in an individual patient. Instead, cerebrovascular state must be inferred from partial observations obtained through multimodal monitoring. Despite the availability of several monitoring approaches, when pressure-, flow-, and volume-based metrics provide conflicting signals, it becomes difficult to determine whether a patient is operating within, approaching, or beyond the limits of autoregulation. This manuscript aims to provide a conceptual framework linking these measurements to their underlying mechanisms to improve interpretation, reconcile apparently discordant findings, and support more informed clinical decision-making.

### Cerebral autoregulation, cerebrovascular reactivity, and pressure reactivity

Cerebrovascular regulation is underpinned by three closely related but physiologically distinct concepts: cerebral autoregulation, cerebrovascular reactivity, and pressure reactivity. Although often used interchangeably, they describe fundamentally different processes. Both concepts have been extensively reviewed, including their underlying mechanisms, assessment methods, and clinical applications [1–7, 9–24]. Therefore, the aim of this manuscript is not to recapitulate the findings in existing reviews, but rather to understand the underlying cerebrovascular mechanisms behind commonly used bedside monitoring indices.

Cerebrovascular reactivity refers to the dynamic responsiveness of the cerebral vasculature to various physiological and pathophysiological stimuli, including fluctuations in arterial carbon dioxide (CO_2_) tension, oxygenation, neural metabolic demand, cerebral blood volume (CaBV), and arterial blood pressure (ABP) [16, 25–27]. It corresponds to the capacity of cerebral vessels to adjust their tone and diameter, influencing both CBF and CaBV. Importantly, cerebrovascular reactivity is not a unitary mechanism but rather denotes a unified response to different stimuli. Two major paradigms are commonly described: pressure reactivity, reflecting myogenic responses to changes in perfusion pressure [28], and chemical/metabolic reactivity, referring to vascular responses to neurogenic, endothelial, and metabolic stimuli, such as CO_2_, pH shifts, or vasoactive drugs [29].

Cerebral autoregulation represents a specific outcome of pressure reactivity: the brain’s intrinsic ability to maintain relatively stable CBF in response to fluctuations in CPP [30, 31]. This mechanism operates effectively within a defined range of CPP, originally depicted by Lassen’s autoregulatory plateau [32], although the concept of a strict CBF plateau and fixed autoregulatory limits has since been challenged due to significant inter- and intra-individual variability [33]. Myogenic responses of cerebral arterioles constitute a major contributor to autoregulation, whereby vascular smooth muscle contracts or relaxes in response to increases or decreases in intraluminal pressure or shear stress, thereby stabilising flow [34, 35].

An expanded methodological overview describing the temporal–spatial dimensions of cerebral autoregulation and cerebrovascular reactivity, together with measurement approaches and commonly used indices, is included in the Supplementary Material S1.

### Bedside assessment of cerebrovascular regulation

While numerous methods are available to assess cerebral autoregulation and cerebrovascular reactivity, continuous bedside monitoring in neurocritical care relies predominantly on correlation-based indices. They quantify intrinsic and spontaneous fluctuations in input (ABP or CPP) and output signals (intracranial pressure – ICP, cerebral blood velocity – CBv, regional oxygen saturation, haemoglobin-derived metrics, brain tissue oxygen tension), negating the need for interventions [36]. Their computational simplicity permits continuous real-time implementation within modern intensive care monitoring systems, producing a single index ranging from − 1 to + 1 that is readily interpreted at the bedside.

Among these, the pressure reactivity index (PRx) [37], mean flow index (Mx) [38], and arterial mean flow index (Mxa) have emerged as key time-domain metrics [39–41]. Their methodological simplicity has facilitated widespread adoption for continuous monitoring in patients with TBI and other forms of acute brain injury [42, 43].

Although these indices are often used as surrogate measures of cerebral autoregulation, they are not interchangeable. Their reported thresholds vary across populations, limiting generalisability [44, 45], and they frequently provide discordant results despite being measured simultaneously. PRx is thought to reflect the combined influence of vascular pressure reactivity and intracranial pressure-volume dynamics, whereas Mx and Mxa are generally interpreted as being more closely related to flow autoregulation. Whether these differences represent distinct physiology or methodological limitations remains uncertain.

### Aim of the review

This review aims to disentangle cerebral autoregulation from cerebrovascular and pressure reactivity by examining the assumptions underlying commonly used monitoring indices and the physiological mechanisms responsible for their behaviour. Particular attention is given to the mechanisms that may cause flow- and volume-based metrics to diverge. To address this challenge, we propose an educational framework linking classical cerebrovascular physiology, described across decades of experimental research, to contemporary multimodal neuromonitoring. We introduce mechanistic physiological pathways to map the dynamic interplay between pressure, flow, volume, and intracranial compliance. Representative examples from TBI are used to illustrate these concepts, as this clinical setting provides the most mature application of continuous monitoring. While this framework is presented within the context of TBI, its potential applicability to other contexts is considered and discussed.

The framework developed in this review builds on three complementary levels of evidence: established physiological observations supported by experimental and clinical studies; mechanistic explanations derived from these observations; and a proposed extension of the classical Lassen autoregulatory curve into a six-zone framework, informed by recent experimental and clinical findings, intended as a conceptual model for organising and interpreting cerebrovascular physiology rather than as a prospectively validated classification.

## The pitfalls of conventional cerebral autoregulation and cerebrovascular reactivity monitoring

Recognising the strengths, limitations, and prerequisites of each method is essential for interpreting cerebrovascular function, distinguishing autoregulatory function from vascular reactivity, and identifying when a metric may be unreliable at the bedside.

### Classical methods

Classical approaches to assessing cerebral autoregulation and cerebrovascular reactivity, most notably the autoregulation index (ARI) and transfer function analysis (TFA), have provided important insights into cerebrovascular physiology and remain widely used in healthy subjects and conditions with relatively preserved intracranial dynamics [46, 47]. These methods model the cerebrovascular system as a linear single-input (ABP), single-output (CBF) control system [4, 17, 48–53]. However, their application in neurocritical care remains limited, as they require high-quality stationary signals, careful signal processing, and linear signal transmission, which often limit their bedside use. Moreover, although consensus statements recommend reporting ICP, they note that incorporating CPP (ABP – ICP) as the input signal into the analytic framework “requires further investigation” [4], despite CPP representing the true pressure gradient driving CBF [54]. As a result, although the ARI and TFA are likely to be optimal under controlled steady-state conditions, their interpretability becomes less straightforward when intracranial dynamics – including intracranial compliance, compensatory reserve, and ICP – are disturbed by pathology [4, 55, 56]. Consequently, they have seen limited adoption in conditions involving significant intracranial dynamics, such as TBI [55–60].

Similar limitations apply to conventional cerebrovascular reactivity assessment. Static CO_2_-reactivity testing, whether performed using functional MRI, transcranial Doppler (TCD), or near-infrared spectroscopy, has proven valuable in evaluating cerebrovascular reserve in cerebrovascular disease and dementia [61, 62]. While these methods quantify vascular responses to vasoactive stimuli, they do not generally account for the accompanying changes in CaBV and ICP predicted by the Monro–Kellie doctrine [63]. This omission is particularly relevant in conditions where intracranial dynamics contribute substantially to pathophysiology. Furthermore, these assessments provide only a limited snapshot of a continuously evolving physiological system and require specialised testing conditions or exogenous stimuli [64].

### Correlation-based metrics

PRx, Mx, and Mxa are frequently conflated, obscuring important differences and may lead to misleading conclusions. Recognising their theoretical and practical differences is essential to ensure their appropriate clinical application. PRx quantifies the ICP response to ABP fluctuations, reflecting changes in CaBV modulated by intracranial compliance and primarily pressure reactivity. In contrast, Mx and Mxa quantify the CBv response to changes in CPP and ABP, respectively, reflecting flow autoregulation. Additionally, Mxa is sometimes described as a non-invasive analogue of PRx or Mx [65–68]. However, Mxa is a distinct index because it assesses CBF responses to ABP regardless of whether these are mediated by ICP, aligning more closely with the classical definition of cerebral autoregulation [32].

PRx is widely used in TBI management where invasive ICP monitoring is routinely available, but its dependence on this measurement limits its broader applicability. In contrast, continuous evaluation of Mx and Mxa requires prolonged, stable TCD measurements of the middle cerebral artery, which are technically more challenging. While extended-duration TCD monitoring is feasible,^69^ its clinical application remains sporadic, limiting the practicality of continuous Mx and Mxa assessment. Moreover, the limited availability of prolonged measurements, particularly in light of the nonstationary mechanism they try to quantify, puts their reproducibility and validity into question [44, 68, 70].

A further limitation common to TCD-derived autoregulatory metrics is that they assess only the insonated vascular territory. Because autoregulatory impairment after TBI may be regionally heterogeneous, measurements obtained from a single cerebral artery may not accurately represent global cerebrovascular regulation, and rely on the assumption that the cross-sectional area of the insonated segment remains relatively constant despite potential myogenic, neurogenic, or metabolic diameter adjustments [71]. Consequently, local autoregulatory findings should be interpreted within the broader clinical and imaging context.

### Assumptions and methodological requirements

Reliable inference of these metrics depends on several methodological requirements and physiological conditions. The principal assumptions, technical requirements, and common confounding factors identified in the literature are summarised below, expanding on recommendations from the CARNet group [5, 13, 22, 72–80].

#### High signal quality and temporal resolution

High-quality, high-resolution recordings of the signals – ABP, ICP, and CBv – are critical to reliably capture the rapid fluctuations in the signals and identify any artefacts [81, 82].

#### Spontaneous ABP variability

Spontaneous slow systemic ABP changes (typically within the frequency range of 0.003–0.05 Hz) are required to drive measurable changes in ICP or CBv, against which the reactivity of the cerebral vasculature and the state of autoregulation are inferred. ABP fluctuations of at least 10% of the mean are recommended for reliable index calculation [72].

#### Monitoring of external confounding factors

Several external and physiological factors can influence ICP and CBv independently of ABP and autoregulatory mechanisms and may mask or mimic autoregulatory impairment. These include technical artefacts (e.g., arterial line failure), pharmacological effects, and nursing procedures (e.g., tracheal suctions, posture adjustments). Physiological variables that can directly modulate vascular tone, ICP, or the pressure-volume relationship – such as changes in posture or body temperature, sympathetic activation, cerebral venous pressure, and breathing frequency – are also sources of confounding [13, 72, 76]. Changes in carbon dioxide and oxygen levels – measured via end-tidal CO_2_ (EtCO_2_), partial pressure of arterial CO_2_, oxygen saturation, or arterial oxygen partial pressure – impact the cerebrovascular tone. Sustained hypercapnia and severe hypoxia promote vasodilation, elevate PRx and Mx values and confound autoregulatory assessment. Fluctuations in CO_2_ and oxygen metrics, if sufficiently rapid, may mimic impaired autoregulation and should therefore be continuously monitored. EtCO_2_ must remain stable within the calculation period for autoregulation metrics to display true pressure-flow relationships [13, 72, 76].

Neuronal activation may trigger neurovascular coupling, a process affecting CBF independently of changes in ABP. Additionally, other nervous responses may arise, such as the Cushing’s response, a reflex caused by high increases in ICP, subsequently impacting peripheral ABP. These effects introduce alternative pathways linking ABP and ICP or CBv, violating the directionality condition (i.e., ABP changes precede and cause ICP/CBv changes). Similarly, cardiac arrhythmias or heart rate variability may produce changes in cardiac output that simultaneously affect mean trends of ABP, CBv, and ICP, mimicking impaired autoregulatory responses.

The transmission of ABP fluctuations to ICP depends on intracranial compliance. When compliance is high, changes in blood volume are not well transmitted to ICP, thereby diminishing the observable coupling between ABP and ICP. In such cases, PRx values must be interpreted with caution. Metrics such as the RAP index, pulse shape index [83, 84], pulsatile skull expansions [85], or TCD-derived compliance indices can provide insight into compliance status, aiding comprehension.

## A unified framework: mechanistic pathways of neurovascular dynamics

### Physiological parameters characterising cerebrovascular and intracranial processes

Effective assessment of multimodal neuromonitoring requires integrating cerebrovascular and intracranial dynamics. A set of key physiological parameters was derived to characterise these interactions [86].

At its core, CPP acts as the net pressure gradient driving CBF, but the resulting flow depends on the balance between this driving force and the opposing forces generated by the cerebral vasculature. This driving force is balanced by vascular resistance, which represents the opposition to flow determined by vascular tone, and arterial wall tension, representing the net smooth muscle forces acting to constrict or relax the vessel wall. Through these mechanisms, the cerebrovascular system continuously adjusts vessel calibre to regulate both CBF and CaBV.

These adjustments are not instantaneous and are constrained by the physical properties of the vascular bed. Vascular compliance describes the physical ability of cerebral vessels to accommodate pressure changes through volume expansion or contraction, thereby linking pressure and volume within the arterial compartment. The operating limits of this system are defined by the critical closing pressure, the minimum ABP required to keep vessels patent, and the diastolic closing margin, which corresponds to the effective perfusion reserve available during diastole. The speed at which the vasculature adapts to changing haemodynamic conditions is captured by the cerebrovascular time constant, integrating both vascular resistance and compliance into a measure of the system’s temporal response.

Because these arterial volume shifts occur within a rigid skull, they are inherently constrained by the surrounding intracranial environment. This dynamic is characterised by intracranial compliance, which describes the change in ICP resulting from a given change in intracranial volume (i.e., the local slope of the pressure-volume curve). A complementary concept is the pressure–volume compensatory reserve, commonly assessed using the RAP index, which denotes the patient’s operating position along the pressure-volume curve and therefore the remaining capacity to accommodate these vascular volume adjustments without triggering dangerous, secondary spikes in ICP. Although preserved compensatory reserve (RAP ≈ 0) is generally associated with good intracranial compliance and exhausted compensatory reserve (RAP ≈ 1 or < 0) with impaired compliance, these concepts are not interchangeable, as the shape and operating point of the pressure-volume curve differ between patients. Detailed definitions and equations are provided in the Supplementary Material S2-B1.

Together, these parameters provide a physiological basis for interpreting cerebrovascular variations and autoregulatory function. As discussed above, the assumption of a stable arterial diameter also underlies these velocity-based TCD metrics – vascular resistance, vascular compliance, and intracranial compliance – and may not hold consistently across patients or prolonged monitoring periods.

**Fig. 1:**
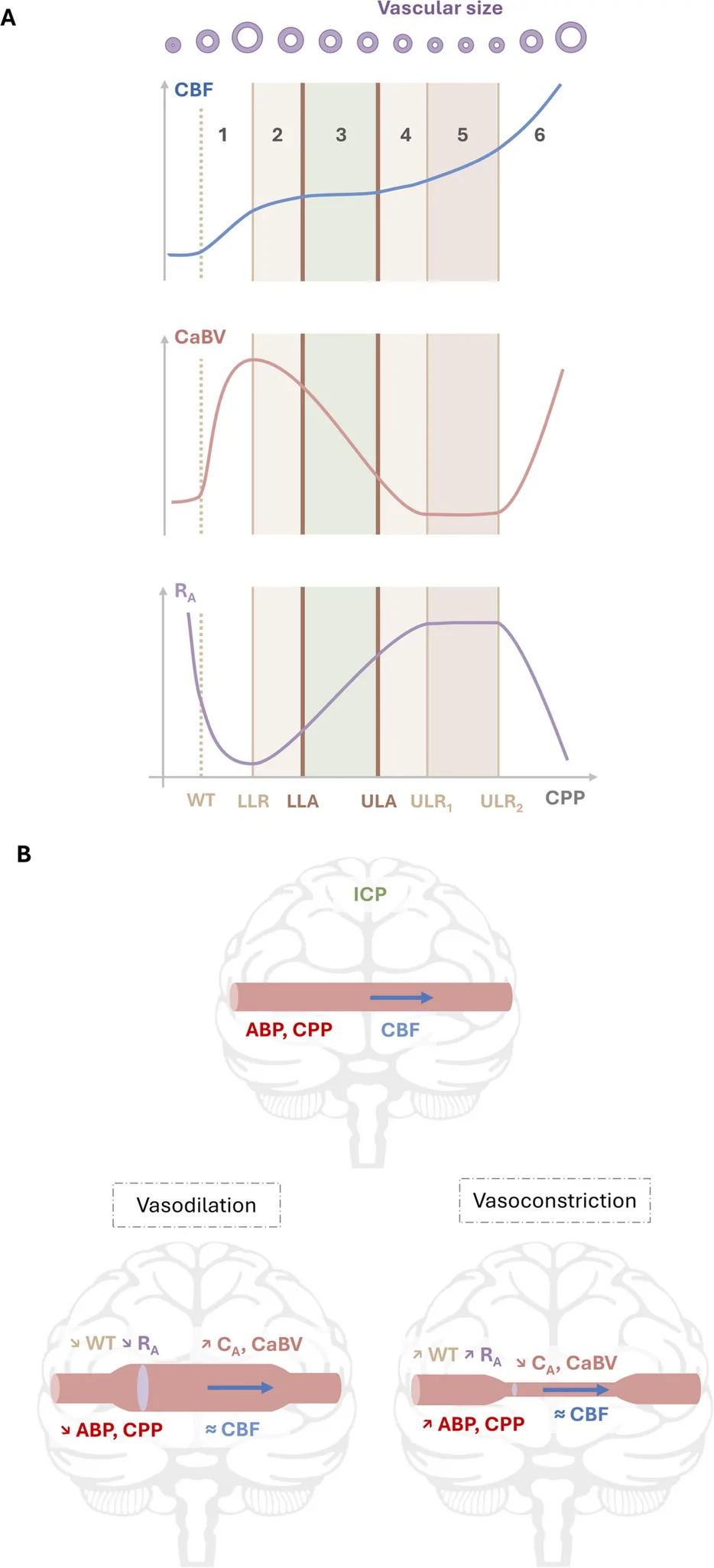
Conceptual relationship between vascular size, cerebral blood flow (CBF), cerebral blood volume (CaBV), vascular resistance (R_A_) and cerebral perfusion pressure (CPP). (**A**) Six phases representing the different states of autoregulation – delimited in part by the lower limit of autoregulation reactivity (LLR), the lower limit of autoregulation (LLA), the upper limits of reactivity (ULRs), and the upper limit of autoregulation (ULA) – are highlighted. The arterial wall tension (WT) is represented to demarcate the critical threshold (CPP = WT) where vessels passively collapse, serving as a direct analogue to the classical critical closing pressure (CrCP) described for arterial blood pressure. The boundaries between phases are not drawn to scale and may vary substantially between individuals. (**B**) Variations in vascular parameters during vasodilation and vasoconstriction. Active vasodilation is characterised by increased compliance (C_A_), increased vascular size, reduced resistance (R_A_), decreased arterial wall tension (WT) and increased CaBV, whereas vasoconstriction exhibits the inverse pattern

Figure 1A illustrates our proposed conceptual model of neurovascular dynamics, integrating classical principles of cerebral circulation physiology with recent experimental evidence [10, 16, 33, 35, 87, 88]. This schematic extends the traditional autoregulatory plateau into six distinct phases to capture the non-linear changes in flow and volume across a full spectrum of CPP. It is intended as a conceptual aid rather than a validated physiological classification. For clarity, we distinguish between two sets of critical pressure limits:

- Lower and upper limits of autoregulation (LLA, ULA) [22, 32]. Within this range, CBF is maintained relatively constant despite changes in CPP through active adjustments in arteriolar diameters, leading to changes in vascular resistance and volume. Beyond, flow becomes pressure-passive.
- Lower and upper limits of reactivity (LLR, ULRs) [89], often wrongly assumed to be identical to LLA and ULA [77, 78, 90], represent the CPP boundaries of working pressure reactivity – i.e., the ability of cerebral vessels to actively respond to CPP changes (vessels constrict with increasing CPP and dilate with decreasing CPP). They lie outside the LLA and ULA, where vascular reactivity can still occur, but CBF has already become pressure-dependent. Beyond the LLR and ULRs, both autoregulation and reactivity are lost, and vascular responses become entirely passive, with vessels either collapsing (CPP < LLR) or passively dilating (CPP > second ULR).

The ABP and CPP ranges associated with each conceptual phase can vary substantially between individuals [91], influenced by factors such as health status, blood gas tensions, anaesthetic exposure, and level of consciousness. Vascular parameters – compliance, resistance, and wall tension – represent myogenic responses linked to changes in vessel diameter and are visualised in Fig. 1B.

### Principles and physiological context

The following mechanistic diagrams are used to visualise cause-and-effect relationships between variables. By formalising dependencies and directions of different influence, these graphs aim to provide a simplified framework to explore the physiological underpinnings of autoregulation and reactivity indices like PRx, Mx, and Mxa, while highlighting conditions under which their prerequisites may be violated. They assume stationary physiological signals, no hidden confounders, and temporal precedence of causal effects. Accordingly, the sign of the correlation-based metrics reflects their expected mathematical behaviour under these ideal conditions rather than clinical thresholds. In clinical practice, overlapping regulatory mechanisms, measurement noise, insufficient ABP excitation, altered intracranial compliance, patient variability and other confounders frequently blur these idealised relationships, leading to higher clinical thresholds, which also increase the certainty of the state identified [37, 92–98].

**Fig. 2:**
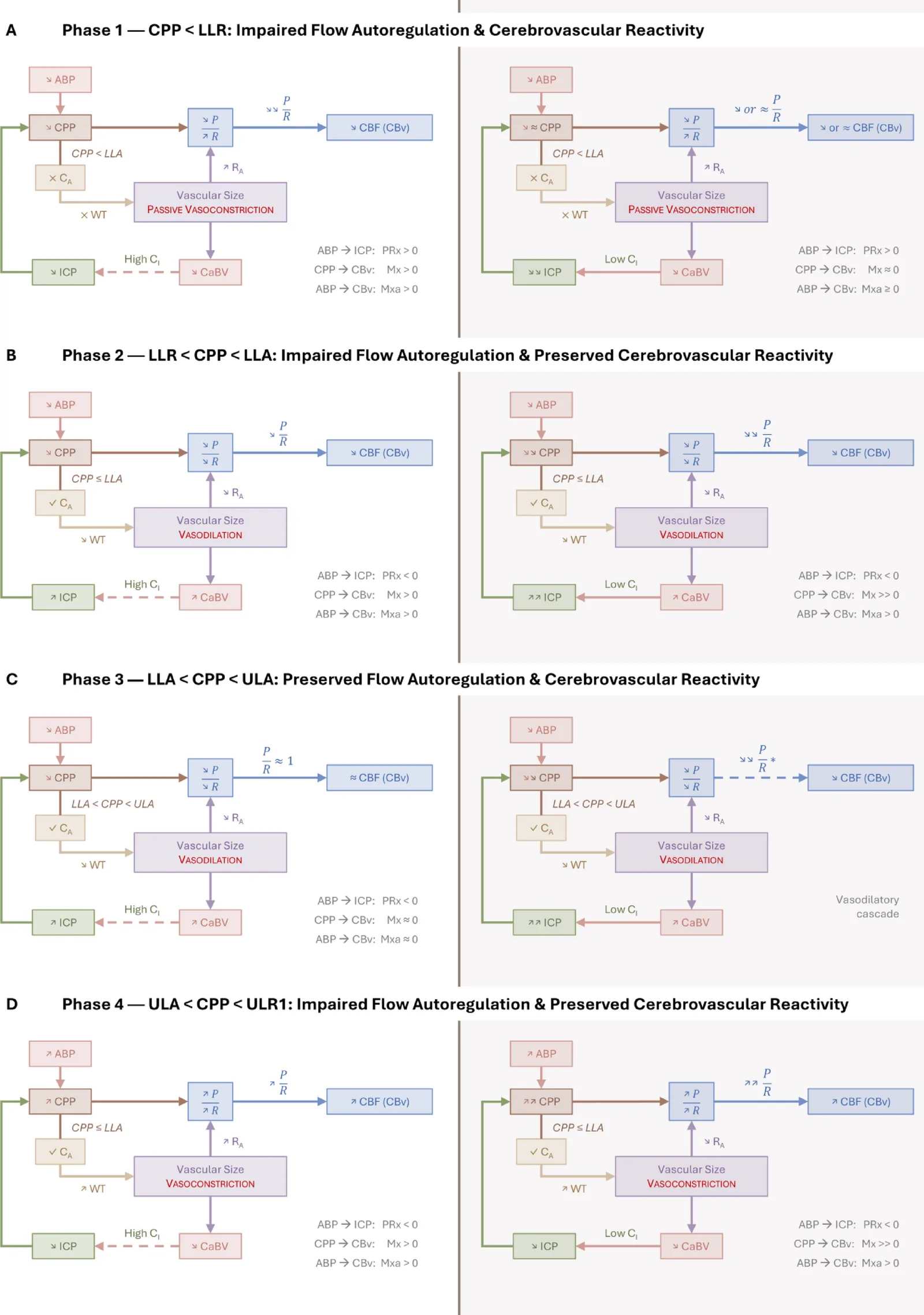
Theoretical mechanistic diagrams interactions among arterial blood pressure (ABP), cerebral perfusion pressure (CPP), cerebral blood flow (CBF) and intracranial pressure (ICP), considering high and low intracranial compliance (C_I_) – Part 1. (**A**) Phase 1: CPP below the lower limit of reactivity (LLR), with an initial decrease in ABP/CPP, leading to passive vasoconstriction, reduced cerebral blood volume (CaBV) and increased in vascular resistance (R_A_), resulting in decreased ICP and CBF. (**B**) Phase 2: CPP between the LLR and lower limit of autoregulation (LLA), with an initial decrease in ABP/CPP inducing vasodilation, increased CaBV, slight reduction in R_A_, increased ICP and decreased CBF. (**C**) Phase 3: CPP within autoregulatory limits, with an initial decrease in ABP/CPP causing vasodilation, increased CaBV and decreased R_A_, visible as increased ICP and maintained (stable) CBF. *In low C_I_ and high ICP conditions, a vasodilatory cascade may occur, further reducing CPP and CBF. (**D**) Phase 4: CPP between the upper limit of autoregulation (ULA) and first upper limit of reactivity (ULR1), with an initial increase in ABP/CPP producing vasoconstriction, reduced CaBV, slight increase in R_A_, visible as decreased ICP and increased CBF

**Fig. 3:**
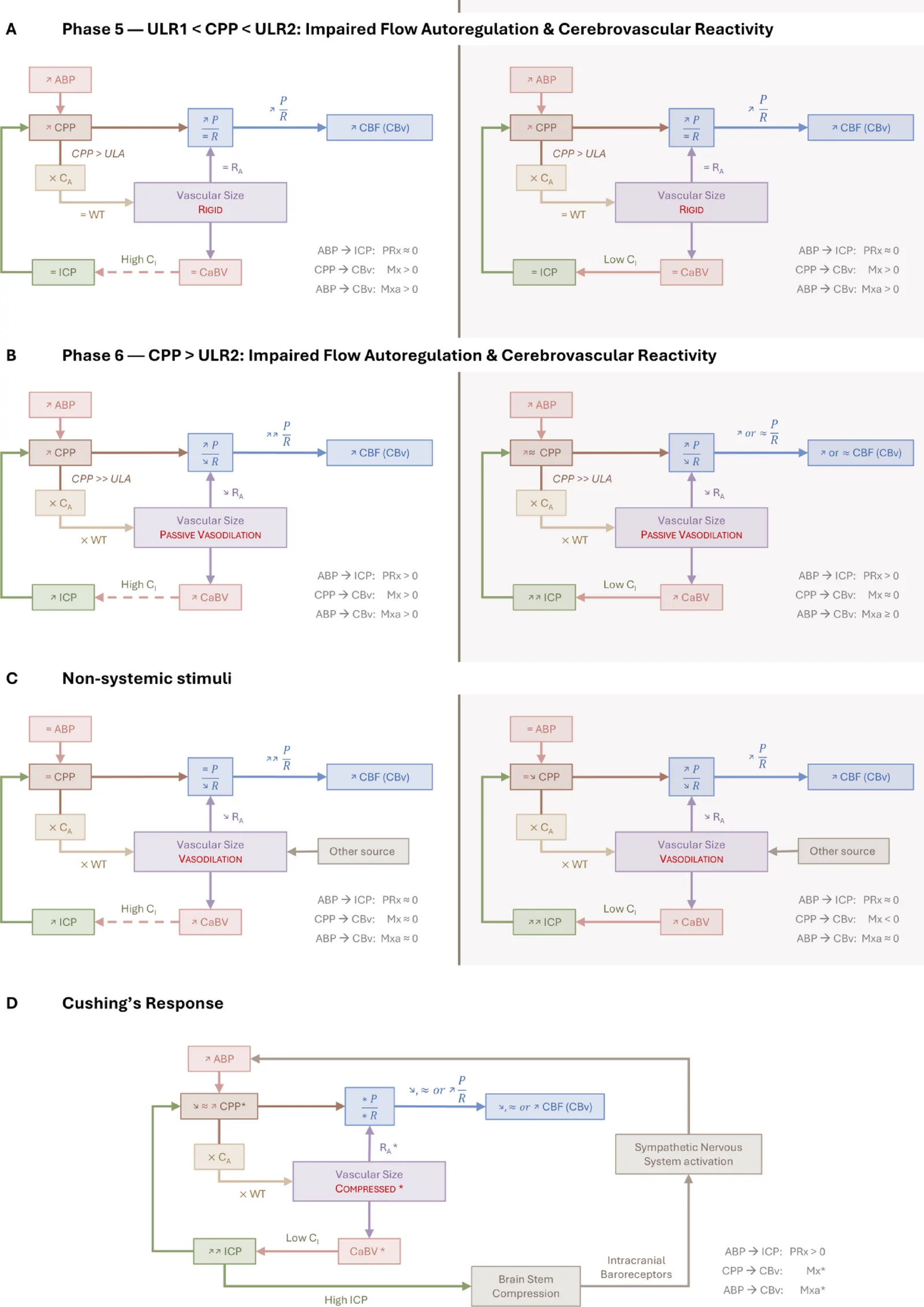
Theoretical mechanistic diagrams among arterial blood pressure (ABP), cerebral perfusion pressure (CPP), cerebral blood flow (CBF), and intracranial pressure (ICP), considering high and low intracranial compliance (C_I_) – Part 2. (**A**) Phase 5: CPP between the upper limits of reactivity (ULRs). Vessels are rigidly constricted; increases in ABP and CPP do not change vessel diameter, cerebral blood volume (CaBV), or vascular resistance (R_A_), resulting in stable ICP and increased CBF. (**B**) Phase 6: CPP above the second ULR. Further ABP/CPP elevation induces passive vasodilation, increasing CaBV and decreasing R_A_, leading to increased ICP and CBF. (**C**) Non-systemic stimuli: External or metabolic influences directly alter vascular tone, here producing vasodilation, increasing CaBV and decreasing R_A_, with consequent rises in ICP and CBF. (**D**) Cushing’s Response: At critically high ICP with reduced C_I_, ICP elevation lowers CPP, triggering passive vasoconstriction and increased R_A_. Brainstem compression activates sympathetic outflow, raising ABP and partially restoring CPP. *Depending on the magnitude of the ABP response, CPP may decrease, stabilise, or increase, producing corresponding decreases, stability, or increases in CBF and autoregulatory indices (Mx/Mxa)

The mechanistic diagrams (Figs. 2 and 3) depict the interactions among key parameters following systemic and local cerebral perturbations. These mechanisms may occur across different states of compensatory reserve. Therefore, RAP was omitted because it describes the patient’s operating position on the pressure-volume curve rather than a component of the pathways themselves. Additionally, RAP is inherently non-linear, with impaired compensatory reserve being described by values closer to 1 or by negative values, which at times average out, leading to perceived adequate compensatory reserve [99]. Paired versions of each diagram are instead shown for distinct levels of intracranial compliance, highlighting how reduced compliance amplifies the effects of volume changes on ICP and, therefore, CPP, whereas preserved compliance is associated with maximal compensation and attenuated pressure fluctuations.

### Phases 1, 5, and 6: Passive vessel and transition states

Phases 1 and 6 (Figs. 2A and 3B) exhibit positive correlations between ABP–CBv (Mxa > 0), CPP–CBv (Mx > 0), and ABP–ICP (PRx > 0), reflecting impaired cerebral autoregulation. In these phases, CPP lies outside the autoregulatory–reactivity range, resulting in compromised myogenic responses. Specifically, when CPP falls below the LLR (Phase 1), vessels that are maximally dilated – with minimal vascular smooth muscle tone – begin to collapse, passively constricted as the intravascular pressure falls below the level needed to maintain vessel patency. In this state, the vessel wall can no longer provide active resistance, resulting first in passive vasoconstriction, followed by complete vasculature collapse once CPP drops below the arterial wall tension (or ABP falls below the critical closing pressure). Conversely, when CPP rises well above the second ULR (Phase 6), vessels already maximally constricted passively dilate as increasing intravascular pressure forces the vessel walls open [87, 100, 101]. These passive vascular states cause corresponding passive changes in ICP and CBv. Low intracranial compliance in these conditions amplifies the counteracting ICP response to changes in CPP, potentially dampening CPP fluctuations. While Mxa and PRx remain largely unchanged, the CPP–CBv correlation (Mx) may decrease toward zero, indicating reduced coupling.

Phase 5 (Fig. 3A) represents a transitional state with CPP between two defined ULRs. Here, vessels are maximally constricted over a range of high CPP due to high smooth muscle tone, resulting in negligible variations in CaBV despite ABP changes [102]. This leads to a near-zero ABP–ICP correlation (PRx ≈ 0) and persistent positive correlations between ABP–CBv and CPP–CBv (Mxa > 0 and Mx > 0). Since volume fluctuations are minimal, low intracranial compliance has little impact on CPP adjustment through ICP feedback. This scenario aligns with experimental observations in piglets and a recent observation in TBI patients, where vessels maintained maximal vasoconstriction over a range of CPP values above the first ULR before passive vasodilation ensued with further CPP increases [87, 88].

### Phases 2 and 4: Active pressure reactivity at autoregulation boundaries

Phases 2 and 4 capture the transition zones between the autoregulatory and reactivity limits, where active vascular reactivity begins to influence cerebral haemodynamics more distinctly.

Phases 2 and 4 (Fig. 2B and D) represent regions between the limits of autoregulation and regulation, a region of “reactivity exhaustion”, where vessels are either maximally dilated (at LLR) or maximally constricted (at the ULRs). In these phases, vascular smooth muscle remains reactive to changes in ABP and CPP, producing inverse changes in CaBV and ICP, reflected by a negative ABP–ICP correlation (PRx < 0). However, this myogenic response is insufficient to fully preserve CBF, with CBv appearing to passively track ABP variations, indicated by positive correlations between ABP–CBv and CPP–CBv (Mxa > 0 and Mx > 0). When intracranial compliance is low, ICP accentuates CPP fluctuations, amplifying changes in vessel diameter, CBF, and ICP. This enhanced coupling is represented in a stronger CPP–CBv correlation, with Mx approaching 1, suggesting a transition toward passive flow–pressure dynamics despite residual autoregulatory activity.

### Phase 3: Intact autoregulation with risk of vasodilatory cascade

Phase 3 (Fig. 2C) represents the region of effective cerebral autoregulation (LLA < CPP < ULA), characterised by a negative correlation between ABP and ICP (PRx < 0), and near-zero correlations between ABP–CBv (Mxa ≈ 0) and CPP–CBv (Mx ≈ 0). In this phase, vascular smooth muscle actively responds to changes in ABP and CPP, causing vasodilation when pressure decreases and vasoconstriction when it increases. This modulation of vessel diameter suppresses fluctuations in blood velocity, introduces a delay due to autoregulatory inertia, and produces an inverse change in ICP. Similar to Phases 2 and 4, low intracranial compliance amplifies CPP variations, resulting in more pronounced ICP changes. Notably, an initial drop in ABP combined with a sharp ICP increase can trigger a vasodilatory cascade that underlies the onset of plateau waves: decreased CPP, exacerbated by rising ICP due to increased CaBV, leads to a drop in CBF and CBv [103]. During such events, PRx remains mostly negative, but Mx and Mxa become positive, though these indices are unreliable due to the unstable nature of this cascade.

Special physiological conditions that may alter the interpretation of mechanistic diagrams, including responses to non-pressure stimuli and ICP–driven reflex mechanisms (e.g., Cushing response), are detailed in the Supplementary Material S2-B2.

## Translating the framework: clinical utility and personalised management

To illustrate the proposed mechanistic model, a series of case examples was selected from a cohort of severe TBI patients undergoing multimodal monitoring [69]. Detailed descriptions of the data acquisition and processing are provided in the Supplementary Material S2-A.

### Patterns of agreement and divergence across autoregulation metrics

#### Case 1: Discordant indices during large ABP swings across autoregulatory limits

##### Case 1

**Fig. 4:**
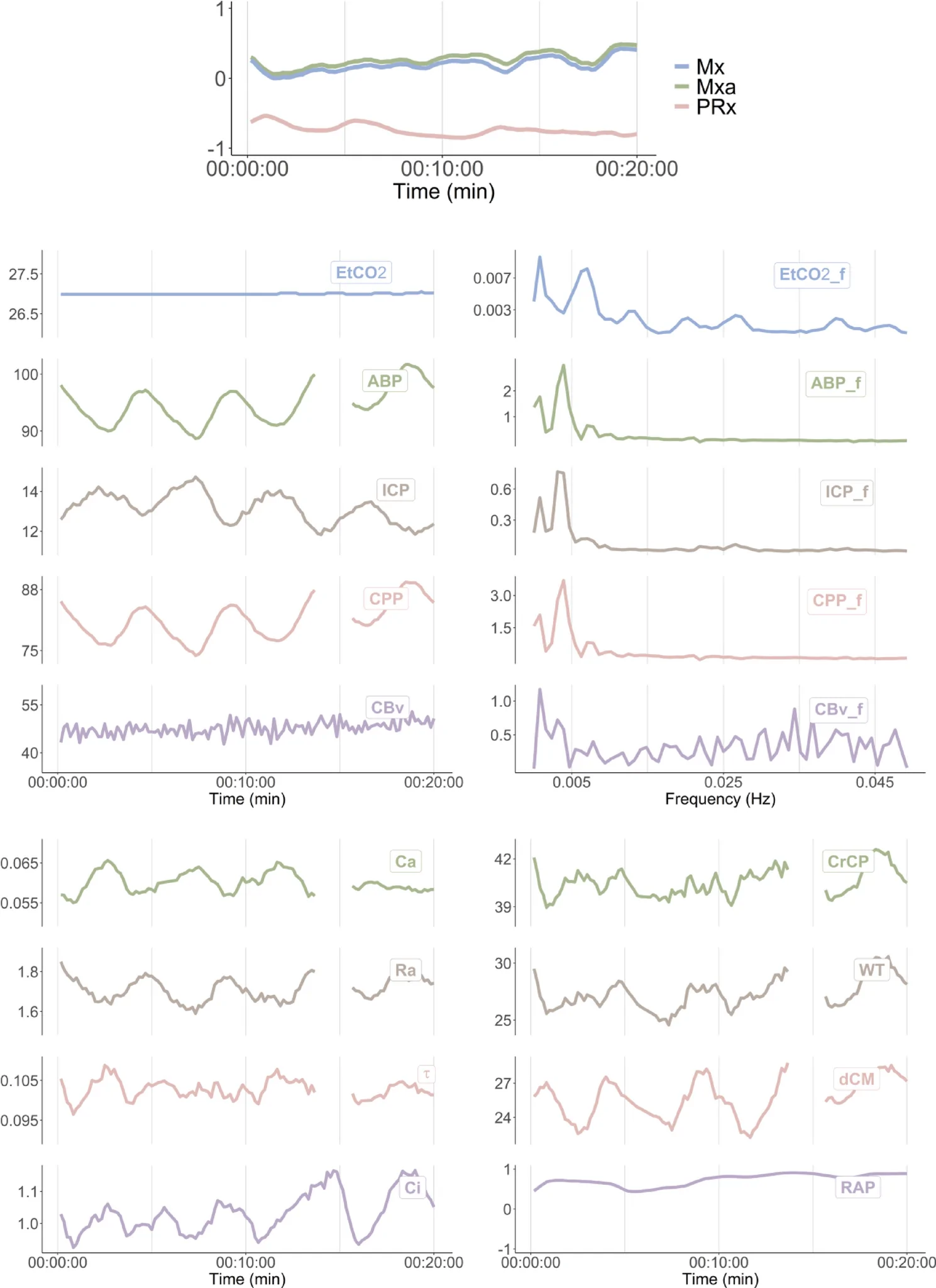
Case 1, representing Phase 2 and 4 (i.e. working cerebrovascular reactivity, impaired cerebral blood flow autoregulation). Significant arterial variations (60–120 mmHg) are observed, leading to subsequent concurrent CPP (40–100 mmHg), EtCO2 (30–32 mmHg) and CBv (50–80 cm/s) fluctuations, but reversed ICP (12–20 mmHg) variations. Ca, Ra and WT are responsive and indicate reactive myogenic responses. Ci is high, with RAP close to 1. ABP: Arterial Blood Pressure; Ca: Arterial compliance; CBv: Cerebral Blood velocity; Ci: Intracranial compliance; CPP: Cerebral Perfusion Pressure; CrCP: Critical Closing Pressure; dCM: Diastolic Closing Margin; EtCO2: End-tidal CO_2_; ICP: Intracranial Pressure; Mx: Mean Flow index; Mxa: Arterial Mean Flow index; PRx: Pressure Reactivity index; Ra: Vascular Resistance; RAP: Pressure-Volume compensatory reserve index; τ: Time constant; WT: Wall Tension; X_f: Frequency chart

demonstrates a striking dissociation between PRx and flow-based metrics (PRx = -1, Mx = Mxa = 1; Fig. 4) during a sedation weaning period. Marked ABP oscillations (60–120 mmHg) induce wide CPP fluctuations (40–100 mmHg), likely traversing both the lower and upper limits of autoregulation.

These ABP variations are mirrored by concurrent changes in CaBV and ICP (12–20 mmHg), with corresponding shifts in vascular resistance, vascular compliance, and arterial wall tension. The vascular response exhibits clear myogenic adaptation: vasodilation during CPP drops and vasoconstriction during rises, consistent with Phases 2 and 4 of the autoregulatory curve (Fig. 2B and D, left panels). As vessel diameter changes, ICP fluctuates accordingly, and intracranial compliance is transiently reduced during ICP elevations, reflecting strain on the compensatory reserve.

Despite preserved pressure reactivity (negative PRx), CBv closely follows ABP due to the high amplitude of pressure oscillations and insufficient counter-regulatory resistance.

This case exemplifies how large, rapid fluctuations in CPP, especially when spanning autoregulatory thresholds, can lead to impaired flow autoregulation, as demonstrated by Mx and Mxa. Despite preserved cerebrovascular pressure reactivity as indicated by PRx, the flow-regulating mechanisms are overwhelmed, highlighting the complementary physiological information provided by PRx, Mx, and Mxa.

Additional representative examples illustrating the remaining patterns of agreement and divergence across autoregulation indices (PRx, Mx, and Mxa) are presented in the Supplementary Material S2-C1.

### Violations of metric assumptions and their consequences

The following case examples depict situations in which the assumptions underlying PRx, Mx, and Mxa are violated, rendering the indices unreliable during these periods.

#### Case 5: Negative Mx in the context of vasogenic oscillations

##### Case 5

**Fig. 5:**
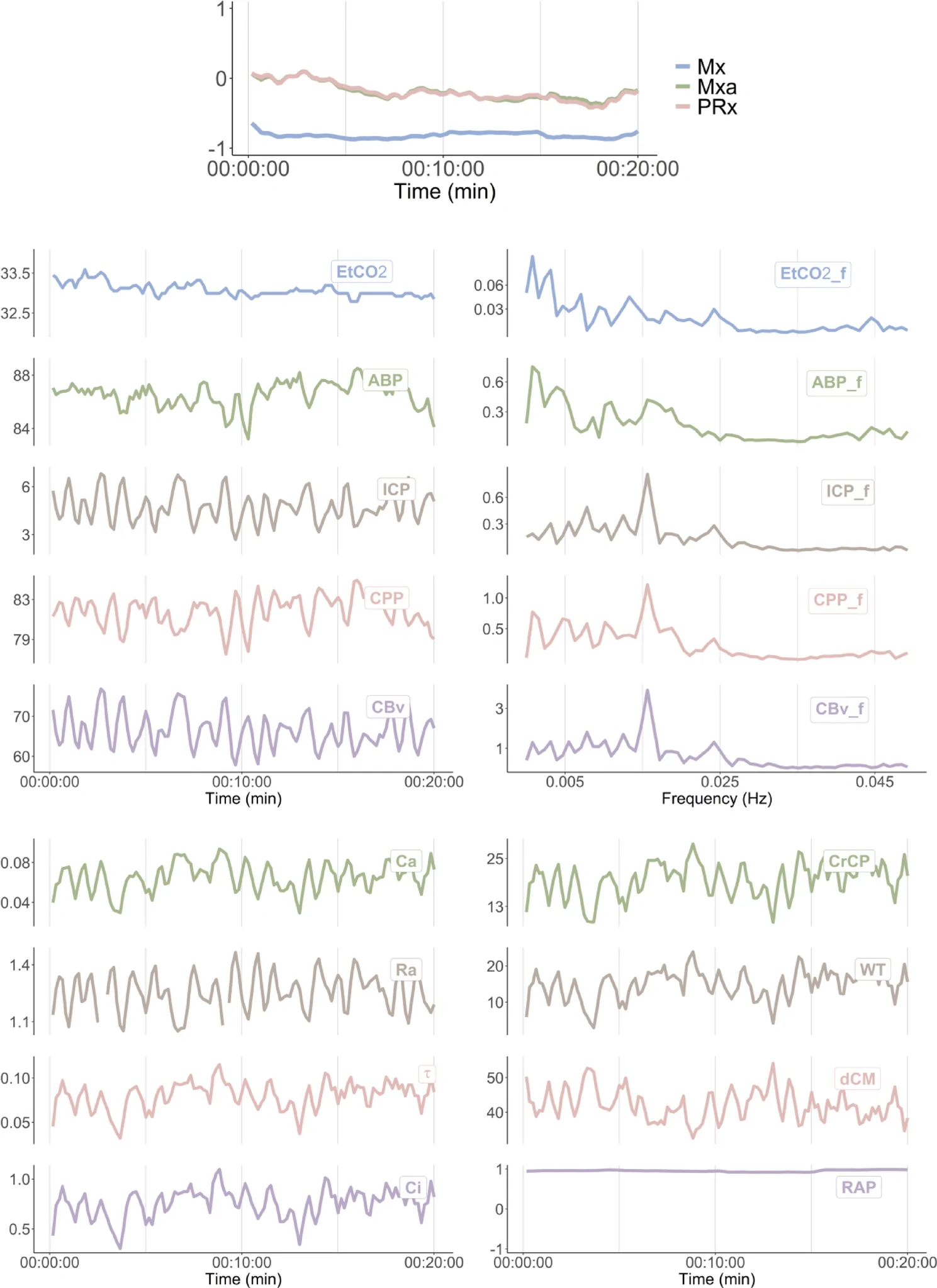
Case 5, representing non-systemic stimuli. Pronounced oscillations (0.015 Hz) are observed in ICP, CPP, and CBv, but not in ABP or EtCO2. Ca and Ci are low, while Ra mirrors the changes seen in ICP and CBv. Mx is particularly negative. ABP: Arterial Blood Pressure; Ca: Arterial compliance; CBv: Cerebral Blood velocity; Ci: Intracranial compliance; CPP: Cerebral Perfusion Pressure; CrCP: Critical Closing Pressure; dCM: Diastolic Closing Margin; EtCO2: End-tidal CO_2_; ICP: Intracranial Pressure; Mx: Mean Flow index; Mxa: Arterial Mean Flow index; PRx: Pressure Reactivity index; Ra: Vascular Resistance; RAP: Pressure-Volume compensatory reserve index; τ: Time constant; WT: Wall Tension; X_f: Frequency chart

(Fig. 5) is characterised by prominent low-frequency oscillations (≈ 0.015 Hz) in ICP, CPP, and CBv, in the absence of corresponding fluctuations in ABP or EtCO_2_. These dynamics suggest that the observed variability originates within the cerebral vasculature itself, rather than being driven by systemic inputs (Fig. 3C, right panel). Vascular compliance is reduced, indicating stiffer vessels that minimise changes in CaBV despite fluctuations in ABP. Compensatory reserve is also diminished, as evidenced by low intracranial compliance, which permits changes in CaBV to propagate directly to ICP. These intracranially generated oscillations in ICP alter CPP, which in turn affects CBv. The resulting inverse relationship between CPP and CBv yields a strongly negative Mx (≈ -1), despite both PRx and Mxa remaining near zero due to the relative stability of ABP and its weak correlation with either ICP or CBv.

Such negative Mx values have previously been interpreted as markers of heightened autoregulatory responsiveness [38, 74, 104], or of non-systemic drivers [86]. In this case, the metrics do not reliably reflect cerebrovascular performance, underscoring the need for cautious evaluation in the presence of endogenously generated vascular oscillations.

#### Case 6: Hypercapnia-induced vasodilation and its confounding effects on autoregulation indices

##### Case 6

**Fig. 6:**
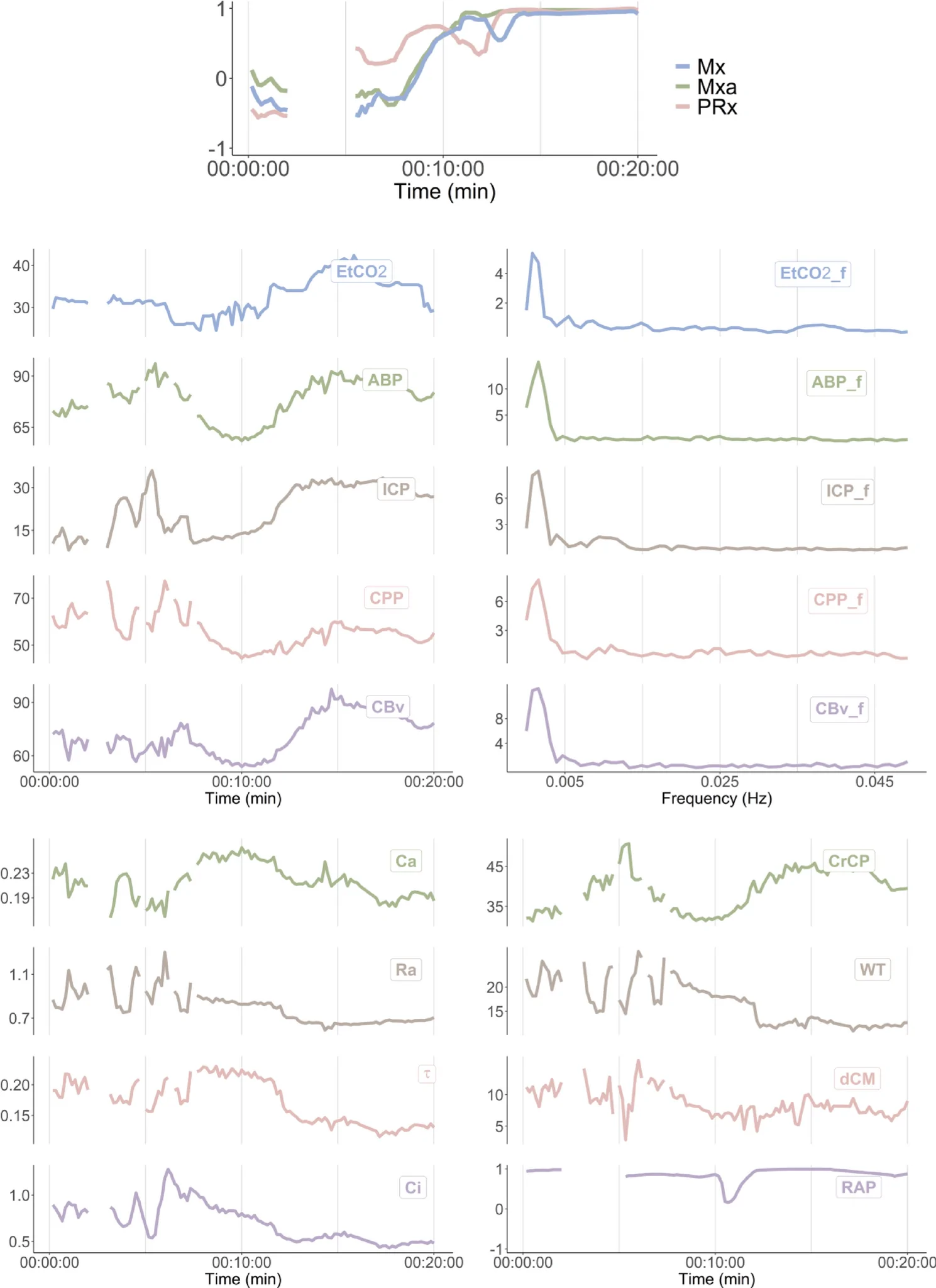
Case 6, representing a violation of PRx and Mx assumptions: CO_2_ variations. A significant rise in EtCO2 is observed (from 25 to 40 mmHg), leading to a passive increase in ABP (60 to 90 mmHg), ICP (10 to 35 mmHg), and CBv (60 to 90 cm/s), alongside a decrease in Ci, Ca, Ra, and WT. As a result, PRx, Mx, and Mxa increase, ultimately reaching a value of 1. ABP: Arterial Blood Pressure; Ca: Arterial compliance; CBv: Cerebral Blood velocity; Ci: Intracranial compliance; CPP: Cerebral Perfusion Pressure; CrCP: Critical Closing Pressure; dCM: Diastolic Closing Margin; EtCO2: End-tidal CO_2_; ICP: Intracranial Pressure; Mx: Mean Flow index; Mxa: Arterial Mean Flow index; PRx: Pressure Reactivity index; Ra: Vascular Resistance; RAP: Pressure-Volume compensatory reserve index; τ: Time constant; WT: Wall Tension; X_f: Frequency chart

(Fig. 6) provides a clear example of the CO_2_ effect on PRx and Mx (Fig. 3C, right panel). A substantial increase in EtCO_2_ (25–40 mmHg) is observed, accompanied by concurrent rises in ICP (10–35 mmHg) and CBv (55–95 cm/s). These changes indicate diffuse vasodilation, as further supported by a reduction in intracranial compliance (indicating reduced compensatory reserve), vascular resistance, and arterial wall tension (suggesting vessel wall relaxation). As a result, PRx, Mx, and Mxa all increase, ultimately reaching values close to 1.

Importantly, while large ABP variations (from 60 to 90 mmHg) would ordinarily favour autoregulatory assessment, the concurrent CO_2_-driven vasodilation violates the requirements underlying these indices. Consequently, elevated PRx, Mx, and Mxa likely do not reflect impaired autoregulatory capacity but rather a strong coupling between CO_2_ content and vessel diameter, underscoring the necessity of accounting for CO_2_ stability when interpreting autoregulatory indices.

#### Case 7: Decompressive craniectomy and the challenge of interpreting flow-based indices

Decompressive craniectomy increases intracranial compliance by allowing cerebral expansion, thereby reducing ICP. It is associated with improved cerebral perfusion and has been reported to partially restore cerebral autoregulation [105, 106]. Timofeev et al. [107] observed a postoperative decrease in RAP – consistent with enhanced compliance – but also a paradoxical rise in PRx, interpreted as a deterioration in cerebrovascular reactivity.

##### Case 7

**Fig. 7:**
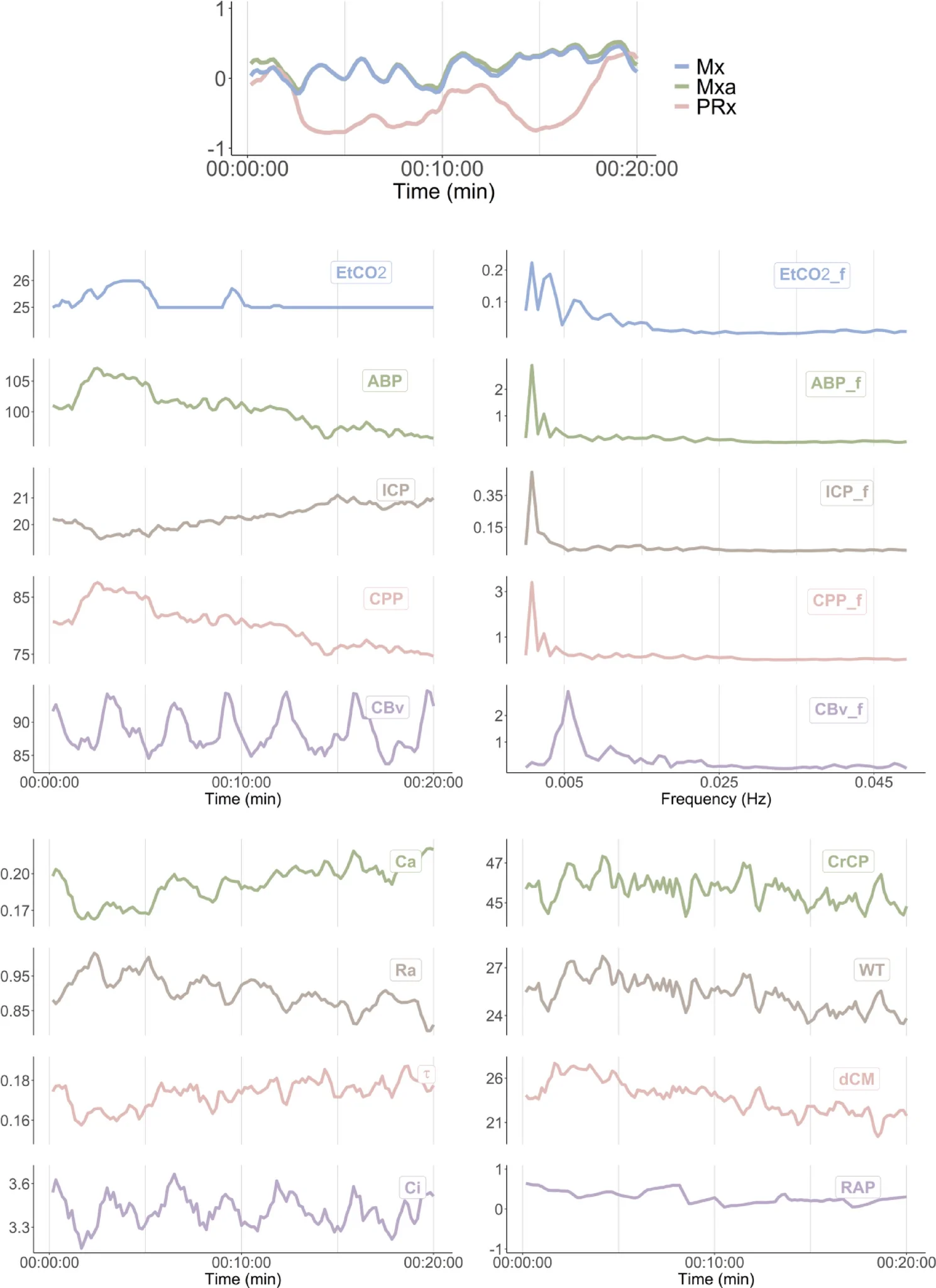
Case 7, representing non-systemic stimuli affecting CBv, with high Ci from decompressive craniectomy. EtCO2 remains constant, while ABP and ICP show long-term variations in opposite directions. CBv displays oscillations at a frequency of 0.005 Hz, which are not observed in ABP or ICP. Ca and Ci are high, with RAP remaining low and close to 0. Ra mirrors changes in CBv, while WT and Ca are displaying large variations corresponding to the ABP changes. Initial and final PRx values close to zero are considered indeterminate as the ABP–ICP relationship is not yet sufficiently established over the correlation window for reliable interpretation. ABP: Arterial Blood Pressure; Ca: Arterial compliance; CBv: Cerebral Blood velocity; Ci: Intracranial compliance; CPP: Cerebral Perfusion Pressure; CrCP: Critical Closing Pressure; dCM: Diastolic Closing Margin; EtCO2: End-tidal CO_2_; ICP: Intracranial Pressure; Mx: Mean Flow index; Mxa: Arterial Mean Flow index; PRx: Pressure Reactivity index; Ra: Vascular Resistance; RAP: Pressure-Volume compensatory reserve index; τ: Time constant; WT: Wall Tension; X_f: Frequency chart

(Fig. 7) was obtained from a patient off sedation following a right-sided decompressive craniectomy performed the previous day. EtCO_2_ remained stable, while ABP decreased from 107 to 95 mmHg and ICP increased from 19 to 22 mmHg, yielding a net decline in CPP. Concurrently, vascular compliance was elevated and rising, and arterial wall tension was reduced, consistent with vasodilation as CPP decreased. High intracranial compliance and low RAP confirmed an augmented compensatory reserve, characteristic of the post-decompressive craniectomy state.

CBv exhibited oscillations at ≈ 0.005 Hz that were absent in ABP or ICP. Vascular resistance showed an overall decreasing trend, matching vasodilation, but also revealed superimposed fluctuations that were inversely related to CBv oscillations. These findings suggest an additional vasogenic driver of flow variability, independent of systemic pressure changes. This local modulation of vessel diameter likely altered CaBV, yet its impact on ICP was muted, likely due to the elevated compliance following decompressive craniectomy.

When interpreted in isolation, PRx, Mx, and Mxa indicate a state consistent with preserved cerebral autoregulation (PRx = -0.5; Mx = Mxa = 0.2; Fig. 2C, left panel). However, the existence of vasogenic oscillations in CBv – unrelated to ABP and confirmed in frequency-domain analysis – casts doubt on the validity of Mx and Mxa, as in Case 5 (Fig. 5). In contrast, PRx may retain interpretive value in this context, since the ABP slow-wave trends are mirrored in ICP, while the faster, locally generated oscillations exert minimal influence on ICP due to high compliance. Finally, the initial and final PRx values near zero should not be interpreted as evidence of preserved or impaired pressure reactivity. Instead, they correspond to an insufficient observation window during which the ABP–ICP relationship cannot yet be reliably estimated. Only after sustained opposing ABP and ICP trends developed did PRx become interpretable as reflecting preserved pressure reactivity.

Additional cases picturing the consequences of altered intracranial compliance are presented in the Supplementary Material S2-C2.

## Conclusions and future directions

This review presents a mechanistic framework for interpreting bedside cerebrovascular monitoring in TBI by linking cerebral autoregulation and cerebrovascular reactivity within a unified physiological model, providing a structured approach to contextualise multimodal monitoring data, even when a patient’s complete autoregulatory curve cannot be directly observed. Maintaining a clear distinction between these processes is essential, as they govern separate physiological pathways and dictate unique clinical consequences.

This framework highlights the importance of continuous assessment of intracranial compliance, a key determinant of the relationship between ABP, ICP, and CBF. In low-compliance states, ICP variations significantly influence CPP, rendering purely ABP-based indices inadequate. Since compliance monitoring currently mostly depends on invasive ICP monitoring, extending its use to other clinical settings where such monitoring is uncommon (e.g., general intensive care, perioperative and resource-limited settings) will likely require two complementary developments. First, reliable non-invasive ICP estimation could help reconstruct the intracranial contribution to CPP and compensatory reserve [108, 109]. Second, non-invasive surrogates of CaBV may permit assessment of pressure reactivity in the absence of ICP monitoring. Potential candidates include low-frequency variations in near-infrared spectroscopy-derived haemoglobin levels [110], and the TCD-derived volume metric [111].

Similarly, although TCD monitoring remains the reference technique for calculating Mx, alternative correlation-based metrics derived from regional cerebral oxygen saturation (COx) [110], cerebral haemoglobin concentration (OHx, THx, DOHx) [36], or brain tissue oxygenation (ORx) [112] have been proposed as surrogate measures. These indices use different input signals, and their generally moderate agreement with Mx suggests that they likely reflect complementary rather than interchangeable aspects of cerebrovascular function [36, 113]. Several of the CBv-based parameters incorporated into the proposed framework may also become more readily accessible from routinely available bedside monitoring. Intracranial compliance can be assessed from ICP wave morphology using indices such as the P2/P1 ratio or the pulse shape index, both based on the progressive increase in the P2 peak as compliance deteriorates [114, 115]. Similarly, the P1 component of the ICP waveform theoretically denotes the transmission of the arterial pulse into the intracranial compartment and may therefore provide information on arterial compliance [116], albeit no validated methodology currently exists. Likewise, optical techniques have demonstrated the feasibility of estimating critical closing pressure, but these approaches remain confined to specialised research settings and would require an additional modality at the bedside [117]. Finally, the wider adoption of continuous CBF monitoring technologies, such as invasive thermal diffusion monitoring (Hemedex) [118], may ultimately enable broader implementation of the physiological framework proposed in this review.

Additionally, applying this conceptual model requires acknowledging two methodological constraints. First, the framework’s mandate for high-resolution, continuous data structures currently restricts its immediate applicability outside highly monitored intensive care environments. Second, the proposed six-phase model remains a conceptual synthesis requiring rigorous experimental and clinical validation to define exact zone thresholds, meaning the included clinical cases serve as educational illustrations of real-world visibility rather than prospective empirical proof of diagnostic accuracy.

Looking forward, optimising patient care will require moving beyond isolated, single-index monitoring toward an integrated, mechanistically grounded understanding of blood flow and volume regulation. Correlation-based indices such as PRx, Mx, and Mxa do not directly measure cerebral autoregulation or cerebrovascular reactivity, but infer these processes from physiological signals under specific methodological assumptions. Consequently, their physiological interpretation depends first on whether these assumptions are reasonably satisfied before attributing meaning to the calculated values. When they hold, discrepancies between indices may provide complementary information reflecting the same physiological mechanisms; if not, discordance may instead indicate unreliable physiological inferences rather than distinct vascular states. Likewise, values close to zero, particularly for PRx, should be interpreted cautiously, as they may indicate either weak physiological coupling or uncertainty arising from violated assumptions.

Because the underlying vascular mechanisms are not specific to TBI, these physiological descriptions may also prove relevant to other acute and non-acute neurological disorders characterised by disturbed intracranial dynamics, particularly as non-invasive surrogates of intracranial and cerebrovascular dynamics continue to evolve. Ultimately, grounding neuromonitoring in shared physiological principles may provide a foundation for future clinical decision-support tools and data-driven approaches, helping to move bedside monitoring beyond descriptive correlations toward more individualised perfusion management.

## Supplementary Information

Below is the link to the electronic supplementary material.

Supplementary Material 1

Supplementary Material 2

## Data Availability

No datasets were generated or analysed during the current study.

## Abbreviations

ABP: Arterial Blood Pressure
ARI: Autoregulation Index
CaBV: Arterial Cerebral Blood Volume
CBF: Cerebral Blood Flow
CBv: Cerebral Blood Velocity
CO_2_: Carbon Dioxide
CPP: Cerebral Perfusion Pressure
EtCO_2_: End-tidal Carbon Dioxide
ICP: Intracranial Pressure
Mx: Mean Flow index
Mxa: Arterial Mean Flow index
PRx: Pressure Reactivity index
RAP: Pressure-volume compensatory reserve index
TBI: Traumatic Brain Injury
TCD: Transcranial Doppler
TFA: Transfer Function Analysis
