# Supplementary material for: Understanding cerebral autoregulation and cerebrovascular reactivity: mechanistic perspectives and clinical insights from TBI
Source: Crit Care. 2026 Aug 4;30:555. doi: 10.1186/s13054-026-06207-2 (PMC13726059; doi:10.1186/s13054-026-06207-2)
Supplement: Supplementary file 1 [file 13054_2026_6207_MOESM1_ESM.pdf]

Defining the Fundamentals: Cerebral Autoregulation vs. Cerebrovascular Reactivity

1. Temporal and Spatial Dimensions

Cerebral autoregulation and cerebrovascular reactivity differ fundamentally in their temporal dynamics and spatial expression, reflecting their distinct underlying mechanisms and stimuli.

Cerebral autoregulation exists along a continuum and operates over different timescales depending on the nature and duration of the stimuli, broadly categorised into two primary mechanisms.[1] Dynamic cerebral autoregulation starts acting within 1 to 5 seconds to buffer sudden changes via fast myogenic and neurogenic mechanisms,[2-4] while static cerebral autoregulation governs slower vascular responses over minutes, engaging structural and metabolic adjustments.[4, 5] This rapid regulation predominantly involves regional vascular territories supplied by specific cerebral arteries, resulting in spatial heterogeneity linked to vascular anatomy.

By comparison, chemical/metabolic cerebrovascular reactivity, particularly responses to arterial carbon dioxide (CO_2) tension, unfolds over a wider temporal spectrum. Initial vasodilatory responses can occur within 5 to 10 seconds, mediated primarily by endothelial signalling and smooth muscle relaxation.[6-8] However, sustained or repetitive stimuli engage slower neurovascular and metabolic pathways, including glial activation and vascular remodelling, influencing blood flow over minutes to hours.[9, 10] Spatially, CO_2 reactivity exhibits pronounced heterogeneity, with stronger responsiveness in cortical grey matter than in white matter regions.[11]

Pressure reactivity, which encompasses vascular responses to changes in blood pressure and intracranial pressure (ICP), overlaps temporally with dynamic cerebral autoregulation by engaging fast myogenic vasomotor responses but also incorporates slower metabolic and structural adaptations.[5, 12] Though often regarded as a global phenomenon, evidence increasingly supports notable regional variation in pressure reactivity, influenced by local differences in vessel density, innervation, and tissue compliance.[13, 14] These spatiotemporal distinctions between chemical/metabolic and pressure reactivity emphasise that, despite both being facets of cerebrovascular responsiveness, they rely on distinct physiological pathways and serve different functional roles.

2. Measurement Domains

Cerebral autoregulation and cerebrovascular reactivity are complex physiological processes that require tailored measurement approaches depending on the stimulus type and temporal-spatial scale. Various methods have been developed to capture these dynamic cerebrovascular responses, ranging from controlled experimental manoeuvres to continuous monitoring indices derived from spontaneous physiological fluctuations.

Cerebral Autoregulation

Static cerebral autoregulation is assessed by examining the sustained relationship between arterial blood pressure (ABP) or cerebral perfusion pressure (CPP) and cerebral blood flow (CBF). Intact static cerebral autoregulation is confirmed if CBF returns to or is maintained at a relatively constant value despite a sustained change in pressure (the characteristic plateau of the Laasen curve); conversely, if CBF changes proportionally to pressure, static cerebral autoregulation is impaired, and the flow is pressure-passive.[15] Sustained pressure changes are commonly produced using several methods. Pharmacological agents, such as vasopressors (e.g., phenylephrine) or vasodilators (e.g., sodium nitroprusside), are used to steadily raise or lower ABP and hold it constant. Mechanical and physiological changes include manoeuvres designed to induce sustained postural or haemodynamic shifts, such as prolonged head-up tilt, lower-body negative pressure, cuff inflation/deflation, or controlled changes in ICP.[1, 3, 15, 16] Two common quantitative approaches are reported for measuring the static cerebral autoregulation state. Percent-change slope computes the percent change in CBF or cerebral blood velocity (CBv) per percent change in CPP or ABP: a slope approaching 0 signifies perfect static cerebral autoregulation (pressure-independent flow), while a slope closer to 1 indicates a passively pressure-flow relationship (impaired static cerebral autoregulation).[3, 15] Static autoregulation index reports the static cerebral autoregulation state by computing the changes in cerebrovascular resistance relative to the change in ABP or CPP.[17, 18] However, a precise, universally accepted algebraic formula for this index is currently lacking in the literature.

Traditional evaluation of dynamic cerebral autoregulation often employs controlled or spontaneous perturbations of ABP. These methodologies are highly developed, constitute the established approach for dynamic cerebral autoregulation assessment, and have been the subject of numerous reviews and consensus white papers.[19, 20] One prominent

example utilising controlled perturbation is the autoregulation index (ARI). This unitless number (ranging from 0 to 9) represents the efficiency of the dynamic cerebral autoregulation response to rapid, induced changes in ABP, such as those caused by a thigh-cuff deflation manoeuvre.[3] The ARI is derived by fitting the measured CBv response to the theoretical Tiecks' model, which provides ten predefined templates of dynamic cerebral autoregulation effectiveness (ARI = 0 denotes passively pressure-dependent flow, and ARI = 9 denotes maximally intact dynamic cerebral autoregulation).

Complementing the ARI are spontaneous fluctuation-based methods, such as Transfer Function Analysis (TFA). TFA is a sophisticated methodology that characterises the frequency-domain relationship between spontaneous oscillations in ABP (input) and CBv (output) to quantify autoregulatory performance across different timescales.[21, 22] TFA yields three primary frequency-dependent parameters, typically assessed in the very low- and low-frequency ranges (0.02-0.2 Hz), which correspond to the main frequency band of dynamic cerebral autoregulation.

- Gain measures the amplitude damping. Intact dynamic cerebral autoregulation is characterised by low gain (CBv fluctuations are damped), whereas impaired dynamic cerebral autoregulation shows high gain (CBv is passively transmitted).
- Phase measures the time delay between the ABP and CBv signals. Intact dynamic cerebral autoregulation exhibits a positive phase (CBv changes lead ABP changes due to the active vascular response), while impaired dynamic cerebral autoregulation has a near-zero or negative phase (ABP and CBv are in sync, reflecting passive transmission).
- Coherence: Measures the linearity and reliability of the relationship. A high coherence is required for the reliable assessment of dynamic cerebral autoregulation integrity.

TFA is highly regarded and constitutes the established methodology for dynamic cerebral autoregulation assessment, formalised through recommendations from the Cerebrovascular Research Network (CARNet).

Chemical/Metabolic Cerebrovascular Reactivity

Unlike assessments of dynamic cerebral autoregulation based on spontaneous pressure fluctuations, cerebrovascular reactivity to metabolic stimuli requires the controlled introduction of a physiological challenge.

The most common approach involves manipulating arterial CO_2 levels, a potent cerebral vasodilator, resulting in a rapid onset and rapid cessation of the effect. Protocols for this challenge include controlled inhalation (e.g., 5% CO_2) or regulating the end-tidal CO_2 ($EtCO_2$) input signal via controlled step changes, clamping, or ramp protocols.[23] While breath-hold challenges have been explored as a low-tech alternative, inconclusive results were reported.[6] Vasoactive pharmacological challenges, such as acetazolamide, are also routinely used to induce known maximal vascular responses and estimate vascular reserve.[24-26] Furthermore, nitric oxide is considered a fundamental and integral molecule in neurovascular coupling from pre-clinical and human studies.[27] Notably, a study in young healthy males has shown that nitric oxide signalling is integral to the neurovascular coupling response: visual stimulation combined with administration of the nitric oxide synthase inhibitor L-NMMA attenuated the hemodynamic response, highlighting a potential neurovascular coupling-based pathway for cerebrovascular reactivity assessment.[28]

The resulting hemodynamic output for both cerebral autoregulation and cerebrovascular reactivity assessment is measured using several high-resolution techniques.[29, 30] Transcranial Doppler (TCD) remains the most widely adopted choice for routine clinical cerebrovascular reactivity assessment, monitoring the resulting changes in mean CBv in major vessels.[31] Near-infrared spectroscopy is another effective non-invasive tool used to assess changes in oxyhaemoglobin and deoxyhaemoglobin concentration, which act as surrogates for changes in CBF and cerebral arterial blood volume (CaBV).[32-35] Alternatively, Magnetic Resonance Imaging (MRI) methods provide crucial regional and spatial information.[36] These include Blood-Oxygen-Level-Dependent (BOLD) functional MRI, which detects changes in microvascular function, as well as Arterial Spin Labelling and Phase-Contrast MRI.[23, 37] Multimodal techniques and comparison between modalities were also explored, providing coverage of different aspects of cerebrovascular reactivity.[30, 38-40]

When a dedicated challenge protocol is used, frequency-domain analysis such as TFA can be applied to the CO_2 input and the corresponding BOLD/TCD output. This goes beyond the simple magnitude measurement of the response (gain) by also quantifying the speed of the vascular reaction (phase) and the reliability of the relationship (coherence) at relevant frequencies.[41-43]

Time-Domain Indices for Autoregulation and Pressure Reactivity

Beyond the established methodologies, a recent review from the CARNet group comprehensively summarised all time-domain methods for dynamic cerebral autoregulation quantification, highlighting the valuable insight brought by analysing the time course of input and output signals.[44] These methods can be broadly categorised into four groups:

- Correlation methods: simple statistical indices that assess the relationship between low-frequency oscillations in the parameters of interest;
- Linear time-invariant systems: models that describe the cerebral vasculature as linear filters, characterised by impulse response modelling to capture the system's dynamic characteristics;
- ARI and Rate of recovery: methods based on mathematical equations fitted to an induced step change in pressure;
- Advanced models: heterogeneous group which includes mathematical compartmental models (which simulate underlying physiological mechanisms), directional sensitivity indices (analysing the differential response to increasing vs. decreasing pressure), multiple-input models, and extensions such as non-linear or time-varying models (which allow linear time-invariant systems parameters to be continuously tracked over time).

The most widely used and commonly applied metrics remain the pressure-reactivity index (PRx), the mean flow index (Mx), and the arterial mean flow index (Mxa). These three indices are correlation-based time-domain metrics that attempt to quantify cerebrovascular responsiveness. Mx quantifies the correlation between spontaneous, slow-wave fluctuations in CPP and CBv.[45, 46] CBv is typically measured non-invasively in the middle cerebral artery using TCD and serves as a proxy measure for CBF, based on the relationship $CBF = CSA \cdot CBv$, where CSA is the cross-sectional area of the middle cerebral artery.[47] For the theoretical Mx definition, the CSA is assumed to be constant, allowing CBv to represent changes in CBF.

Mxa is closely related to Mx, but measures the correlation between ABP and CBv, without incorporating ICP. Unlike Mx, which depends on CPP, Mxa assesses autoregulatory capacity solely based on ABP-induced changes in flow, offering a distinct

window into classical cerebral autoregulation mechanisms by removing the influence of intracranial compliance and pressure dynamics.

Complementing these, PRx is derived by correlating slow-wave oscillations of ABP and ICP.[12] It serves as a global index of cerebrovascular pressure reactivity, reflecting blood volume changes brought by cerebral vessel dilatation or constriction in response to changes in ABP.[48] PRx, therefore, reflects the changes in CaBV in response to changes in cerebral vessels' size, a product of cerebral autoregulation, and is widely used clinically to monitor autoregulatory function in patients with brain injury.[12, 49]

Together, these diverse methods capture different facets of cerebrovascular physiology: from controlled perturbations versus spontaneous fluctuations, and chemical versus pressure stimuli, to regional versus global vascular responses.

References

1. Claassen J, Thijssen DHJ, Panerai RB, Faraci FM. Regulation of cerebral blood flow in humans: physiology and clinical implications of autoregulation. Physiol Rev. 2021;101(4):1487-559.

2. Aaslid R, Lindegaard KF, Sorteberg W, Nornes H. Cerebral autoregulation dynamics in humans. Stroke. 1989;20(1):45-52.

3. Tiecks FP, Lam AM, Aaslid R, Newell DW. Comparison of static and dynamic cerebral autoregulation measurements. Stroke. 1995;26(6):1014-9.

4. Panerai RB. Assessment of cerebral pressure autoregulation in humans--a review of measurement methods. Physiol Meas. 1998;19(3):305-38.

5. Lassen NA. Cerebral blood flow and oxygen consumption in man. Physiol Rev. 1959;39(2):183-238.

6. Bright MG, Murphy K. Reliable quantification of BOLD fMRI cerebrovascular reactivity despite poor breath-hold performance. Neuroimage. 2013;83:559-68.

7. Tancredi FB, Hoge RD. Comparison of cerebral vascular reactivity measures obtained using breath-holding and CO2 inhalation. J Cereb Blood Flow Metab. 2013;33(7):1066-74.

8. Willie CK, Tzeng YC, Fisher JA, Ainslie PN. Integrative regulation of human brain blood flow. J Physiol. 2014;592(5):841-59.

9. Attwell D, Buchan AM, Charpak S, Lauritzen M, Macvicar BA, Newman EA. Glial and neuronal control of brain blood flow. Nature. 2010;468(7321):232-43.

10. Iadecola C. The Neurovascular Unit Coming of Age: A Journey through Neurovascular Coupling in Health and Disease. Neuron. 2017;96(1):17-42.

11. Fierstra J, Sobczyk O, Battisti-Charbonney A, Mandell DM, Poublanc J, Crawley AP, et al. Measuring cerebrovascular reactivity: what stimulus to use? J Physiol. 2013;591(23):5809-21.

12. Czosnyka M, Smielewski P, Kirkpatrick P, Laing RJ, Menon D, Pickard JD. Continuous assessment of the cerebral vasomotor reactivity in head injury. Neurosurgery. 1997;41(1):11-7; discussion 7-9.

13. Toth P, Tarantini S, Csiszar A, Ungvari Z. Functional vascular contributions to cognitive impairment and dementia: mechanisms and consequences of cerebral autoregulatory dysfunction, endothelial impairment, and neurovascular uncoupling in aging. Am J Physiol Heart Circ Physiol. 2017;312(1):H1-H20.

14. Sainbhi AS, Marquez I, Gomez A, Stein KY, Amenta F, Vakitbilir N, et al. Regional disparity in continuously measured time-domain cerebrovascular reactivity indices: a scoping review of human literature. Physiol Meas. 2023;44(7):07TR2.

15. Fantini S, Sassaroli A, Tgavalekos KT, Kornbluth J. Cerebral blood flow and autoregulation: current measurement techniques and prospects for noninvasive optical methods. Neurophotonics. 2016;3(3):031411.

16. Ruesch A, Acharya D, Schmitt S, Yang J, Smith MA, Kainerstorfer JM. Comparison of static and dynamic cerebral autoregulation under anesthesia influence in a controlled animal model. PLoS One. 2021;16(1):e0245291.

17. Preisman S, Marks R, Nahtomi-Shick O, Sidi A. Preservation of static and dynamic cerebral autoregulation after mild hypothermic cardiopulmonary bypass. Br J Anaesth. 2005;95(2):207-11.

18. Steiner LA, Coles JP, Johnston AJ, Chatfield DA, Smielewski P, Fryer TD, et al. Assessment of cerebrovascular autoregulation in head-injured patients: a validation study. Stroke. 2003;34(10):2404-9.

19. Claassen JA, Meel-van den Abeelen AS, Simpson DM, Panerai RB, international Cerebral Autoregulation Research N. Transfer function analysis of dynamic cerebral autoregulation: A white paper from the International Cerebral Autoregulation Research Network. J Cereb Blood Flow Metab. 2016;36(4):665-80.

20. Panerai RB, Brassard P, Burma JS, Castro P, Claassen JA, van Lieshout JJ, et al. Transfer function analysis of dynamic cerebral autoregulation: A CARNet white paper 2022 update. J Cereb Blood Flow Metab. 2023;43(1):3-25.

21. Zhang R, Zuckerman JH, Giller CA, Levine BD. Transfer function analysis of dynamic cerebral autoregulation in humans. Am J Physiol. 1998;274(1 Pt 2):H233-41.

22. Meel-van den Abeelen AS, Simpson DM, Wang LJ, Slump CH, Zhang R, Tarumi T, et al. Between-centre variability in transfer function analysis, a widely used method for linear quantification of the dynamic pressure-flow relation: the CARNet study. Med Eng Phys. 2014;36(5):620-7.

23. Liu P, De Vis JB, Lu H. Cerebrovascular reactivity (CVR) MRI with CO2 challenge: A technical review. Neuroimage. 2019;187:104-15.

24. Settakis G, Molnar C, Kerenyi L, Kollar J, Legemate D, Csiba L, et al. Acetazolamide as a vasodilatory stimulus in cerebrovascular diseases and in conditions affecting the cerebral vasculature. Eur J Neurol. 2003;10(6):609-20.

25. Wu J, Dehkharghani S, Nahab F, Qiu D. Acetazolamide-augmented dynamic BOLD (aczBOLD) imaging for assessing cerebrovascular reactivity in chronic steno-occlusive disease of the anterior circulation: An initial experience. Neuroimage Clin. 2017;13:116-22.

26. Dogra S, Wang X, Gupta A, Veraart J, Ishida K, Qiu D, et al. Acetazolamide-augmented BOLD MRI to Assess Whole-Brain Cerebrovascular Reactivity in Chronic Steno-occlusive Disease Using Principal Component Analysis. Radiology. 2023;307(3):e221473.

27. Scarpellino G, Brunetti V, Berra-Romani R, De Sarro G, Guerra G, Soda T, et al. The Unexpected Role of the Endothelial Nitric Oxide Synthase at the Neurovascular Unit: Beyond the Regulation of Cerebral Blood Flow. Int J Mol Sci. 2024;25(16):9071.

28. Hoiland RL, Caldwell HG, Howe CA, Nowak-Fluck D, Stacey BS, Bailey DM, et al. Nitric oxide is fundamental to neurovascular coupling in humans. J Physiol. 2020;598(21):4927-39.

29. Sainbhi AS, Gomez A, Froese L, Slack T, Batson C, Stein KY, et al. Non-Invasive and Minimally-Invasive Cerebral Autoregulation Assessment: A Narrative Review of Techniques and Implications for Clinical Research. Front Neurol. 2022;13:872731.

30. Zhong J, Li G, Lv Z, Chen J, Wang C, Shao A, et al. Neuromodulation of Cerebral Blood Flow: A Physiological Mechanism and Methodological Review of Neurovascular Coupling. Bioengineering (Basel). 2025;12(5):442.

31. Willie CK, Colino FL, Bailey DM, Tzeng YC, Binsted G, Jones LW, et al. Utility of transcranial Doppler ultrasound for the integrative assessment of cerebrovascular function. J Neurosci Methods. 2011;196(2):221-37.

32. Kato T, Kamei A, Takashima S, Ozaki T. Human visual cortical function during photic stimulation monitoring by means of near-infrared spectroscopy. J Cereb Blood Flow Metab. 1993;13(3):516-20.

33. Ward LM, Aitchison RT, Tawse M, Simmers AJ, Shahani U. Reduced Haemodynamic Response in the Ageing Visual Cortex Measured by Absolute fNIRS. PLoS One. 2015;10(4):e0125012.

34. Miller S, Mitra K. NIRS-based cerebrovascular regulation assessment: exercise and cerebrovascular reactivity. Neurophotonics. 2017;4(4):041503.

35. Amyot F, Davis C, Sangobowale M, Moore C, Silverman E, Gandjbakhche A, et al. Cerebrovascular Reactivity Measurement with Functional Near Infrared Spectroscopy: 1940-087X; 2022. e61284 p.

36. Sleight E, Stringer MS, Marshall I, Wardlaw JM, Thrippleton MJ. Cerebrovascular Reactivity Measurement Using Magnetic Resonance Imaging: A Systematic Review. Front Physiol. 2021;12:643468.

37. Sobczyk O, Battisti-Charbonney A, Fierstra J, Mandell DM, Poublanc J, Crawley AP, et al. A conceptual model for CO(2)-induced redistribution of cerebral blood flow with experimental confirmation using BOLD MRI. Neuroimage. 2014;92:56-68.

38. Totaro R, Barattelli G, Quaresima V, Carolei A, Ferrari M. Evaluation of potential factors affecting the measurement of cerebrovascular reactivity by near-infrared spectroscopy. Clin Sci (Lond). 1998;95(4):497-504.

39. Tong Y, Bergethon PR, Frederick BD. An improved method for mapping cerebrovascular reserve using concurrent fMRI and near-infrared spectroscopy with Regressor Interpolation at Progressive Time Delays (RIPTiDe). Neuroimage. 2011;56(4):2047-57.

40. Burley CV, Francis ST, Thomas KN, Whittaker AC, Lucas SJE, Mullinger KJ. Contrasting Measures of Cerebrovascular Reactivity Between MRI and Doppler: A Cross-Sectional Study of Younger and Older Healthy Individuals. Front Physiol. 2021;12:656746.

41. Carrera E, Lee LK, Giannopoulos S, Marshall RS. Cerebrovascular reactivity and cerebral autoregulation in normal subjects. J Neurol Sci. 2009;285(1-2):191-4.

42. Regan RE, Fisher JA, Duffin J. Factors affecting the determination of cerebrovascular reactivity. Brain Behav. 2014;4(5):775-88.

43. Sayin ES, Sobczyk O, Poublanc J, Mikulis DJ, Fisher JA, Kuo KHM, et al. Assessment of cerebrovascular function in patients with sickle cell disease using transfer function analysis. Physiol Rep. 2022;10(19):e15472.

44. Kostoglou K, Bello-Robles F, Brassard P, Chacon M, Claassen JA, Czosnyka M, et al. Time-domain methods for quantifying dynamic cerebral blood flow autoregulation:

Review and recommendations. A white paper from the Cerebrovascular Research Network (CARNet). J Cereb Blood Flow Metab. 2024;44(9):1480-514.

45. Czosnyka M, Smielewski P, Kirkpatrick P, Menon DK, Pickard JD. Monitoring of cerebral autoregulation in head-injured patients. Stroke. 1996;27(10):1829-34.

46. Czosnyka M, Smielewski P, Lavinio A, Pickard JD, Panerai R. An assessment of dynamic autoregulation from spontaneous fluctuations of cerebral blood flow velocity: a comparison of two models, index of autoregulation and mean flow index. Anesth Analg. 2008;106(1):234-9, table of contents.

47. Skow RJ, Brothers RM, Claassen J, Day TA, Rickards CA, Smirl JD, et al. On the use and misuse of cerebral hemodynamics terminology using transcranial Doppler ultrasound: a call for standardization. Am J Physiol Heart Circ Physiol. 2022;323(2):H350-H7.

48. Klein SP, Decraene B, De Sloovere V, Kempen B, Meyfroidt G, Depreitere B. The Pressure Reactivity Index as a Measure for Cerebrovascular Autoregulation: Validation in a Porcine Cranial Window Model. Neurosurgery. 2024;95(6):1450-6.

49. Aries MJ, Czosnyka M, Budohoski KP, Steiner LA, Lavinio A, Kolias AG, et al. Continuous determination of optimal cerebral perfusion pressure in traumatic brain injury. Crit Care Med. 2012;40(8):2456-63.
