# Supplementary material for: Understanding cerebral autoregulation and cerebrovascular reactivity: mechanistic perspectives and clinical insights from TBI
Source: Crit Care. 2026 Aug 4;30:555. doi: 10.1186/s13054-026-06207-2 (PMC13726059; doi:10.1186/s13054-026-06207-2)
Supplement: Supplementary file 2 [file 13054_2026_6207_MOESM2_ESM.pdf]

A – Data Acquisition and Processing

To provide context for the presented case examples, this section details the patient cohort, ethical considerations, monitoring methods, and data processing techniques employed.

1. Ethics

Deidentified data were retrieved from the Brain Physics database under ethical approval (REC 23/YH/0085). Transcranial Doppler (TCD) monitoring was carried out as part of a clinical audit (Clinical Project ID4201: TCD-based assessment of intracranial haemodynamics in patients admitted to NCCU).[1] Patient characteristics, including sex, age, and Glasgow Coma Scale scores, were collected.

2. Multimodal Monitoring

Intracranial pressure (ICP) was monitored using an intraparenchymal pressure sensor (Codman ICP MicroSensor® (Codman & Shurtleff, Massachusetts, US)). Arterial blood pressure (ABP) was measured using intra-arterial catheters inserted into the radial or femoral artery and zeroed to the level of the third ventricle (Arterial line, Transpac® IV Pressure Transducer (Baxter Healthcare, Illinois, US)). End-tidal carbon dioxide ($EtCO_2$) was assessed using mainstream capnographs integrated into the GE CARESCAPE™ Respiratory Modules E-sCO(V) (GE Healthcare Technologies, Illinois, US). TCD probes were applied bilaterally and fixed in position using a probe holder for a prolonged insonation of the middle cerebral artery (MCA). The devices used were the Doppler Box® (DWL Compumedics, Singen, Germany) or the Delica EMS 9D System® (Shenzen Delica Medical Equipment Co. Ltd, China).

All signals of interest (ABP, ICP, bilateral cerebral blood velocity [CBv] and $EtCO_2$) were integrated using the ICM+® software (Cambridge Enterprise Ltd, Cambridge, UK),[2] recorded with the highest available frequency and then resampled to a sampling frequency of 240 Hz.

3. Signal preprocessing

All the data cleaning and signal processing were performed using ICM+®. Through visual inspections, only a single CBv trace of better quality of the two was chosen for analysis. High-resolution (raw waveform traces) data were processed by experienced clinicians to mark for exclusion segments of non-physiologic or highly distorted waveforms. Those included low signal-to-noise ratio in CBv, arterial line clogging and flushing, and

movement-related artefacts. In addition, automated cleaning was performed for both ABP and ICP, which excluded values outside of the physiological range (i.e., ABP > 300 mmHg or < 0 or ABP pulse amplitude of < 20 mmHg; ICP of <−30 or >200 mmHg) as well as flat lines (signals with no variations).[1]

Pulsatile cerebral blood volume (CaBV) was derived from the high-resolution CBv waveforms by integration over time around its beat mean value.[3] For the subsequent calculations, ABP, ICP, $EtCO_2$, CBv and CaBV were downsampled to 0.1 Hz.

4. Physiological parameters explored

In addition to time trends of the raw parameters, we explored various additional metrics describing the cerebrovascular properties. Amplitude spectra of $EtCO_2$, ABP, ICP, CPP, and CBv were computed using a fast-Fourier transform and plotted over the 0 to 0.05 Hz range. The calculation of the autoregulation indices was based on the previously described methodology.[4] Mx was calculated as the moving Pearson correlation coefficient between CPP and CBv, from a window of 30 consecutive samples, with an update rate of 1 sample (10s). Similarly, PRx was calculated as the moving Pearson correlation coefficient between ABP and ICP. Additionally, the Mxa was calculated similarly to Mx, using ABP rather than CPP.

B – A Unified Framework: The Causal Models of Neurovascular Dynamics

1. Physiological Parameters Characterising Cerebrovascular and Intracranial Dynamics

Table S1. Formula and description of the physiological parameters. *ABP*: Arterial blood pressure; *aABP*: Arterial blood pressure fundamental pulse harmonic amplitude; *aCaBV*: Cerebral blood volume fundamental pulse harmonic amplitude; *AMP*: Intracranial pressure fundamental pulse harmonic amplitude; *dABP*: Diastolic ABP; *CBv*: Cerebral blood velocity; *HR*: Heart Rate; *ICP*: Intracranial pressure.

Parameters	Formula	Description
**CPP:** Cerebral perfusion pressure	$CPP = ABP - ICP$	Pressure gradient driving blood flow to the brain.
**CaBV:** Cerebral arterial blood volume[3]	$CaBV = \int_{t_0}^{t_1} (CBv - \overline{CBv})dt$	Approximation of changes in blood volume within the cerebral arterial vascular bed, derived from CBv waveform
**C_A:** Vascular compliance[5]	$C_A = \frac{aCaBV}{aABP}$	Ability of blood to accommodate changes in ABP through corresponding changes in CaBV, with higher values indicating a more distensible vascular system capable of mitigating pressure fluctuations and maintaining stable perfusion.
**R_A:** Vascular resistance[5]	$R_A = \frac{CPP}{CBv}$	Opposition within the circulatory system that regulates blood pressure and blood flow, reflecting both intrinsic vascular tone and extrinsic factors influencing perfusion.
**CrCP:** Critical closing pressure[5]	$CrCP = ABP - \frac{CPP}{\sqrt{(R_A \cdot C_A \cdot HR \cdot 2\pi)^2 + 1}}$	Minimum pressure required to maintain vessel patency and prevent collapse. Below this threshold, blood vessels collapse due to the tension in the arterial walls overcoming the transmural pressure.
**WT:** Wall tension[5]	$WT = CrCP - ICP$	Net radial force acting on the vessel walls, and integrates forces derived from both passive elastic properties and active smooth muscle tone. Sensitive to changes in both perfusion pressure and intracranial spatial constraint
**τ:** Time constant[6]	$\tau = C_A \cdot R_A$	Indicator of cerebrovascular system temporal responsiveness to perturbations. Higher values suggest a more stable and buffered system with slower haemodynamic transitions, whereas a shorter time constant may indicate rapid and potentially unstable responses.

**dCM:** Diastolic closing margin[7]	$dCM = dABP - CrCP$	Marker of effective cerebral perfusion during diastole. Low or negative values, particularly in the context of raised ICP, signal increased vulnerability to cerebral hypoperfusion and potential ischaemia.
**C_I:** Intracranial compliance[8, 9]	$C_I = \frac{aCaBV}{AMP}$	Capacity of the intracranial compartment to accommodate volume increases without marked rises in ICP. High values reflect a more adaptive intracranial system capable of compensating for volume shifts, whereas lower values are observed in extreme pathological states such as cerebral oedema or space-occupying lesions.
**RAP:** Pressure-volume compensatory reserve index[9, 10]	Correlation coefficient between ICP and AMP, over a 300s window, with a 10s update.	Depiction of the reserve capacity of the intracranial system to buffer volume changes, via cerebrospinal fluid redistribution or venous outflow. A value near 0 suggests a robust pressure-volume compensatory reserve, while a value near 1 indicates poor reserve. Negative values are indicative of vessel collapse.

2. Causal Plots: Special Cases

Cerebrovascular Responses to Other Stimuli

Other mechanisms beyond ABP fluctuations can influence the cerebrovascular bed (Fig. 3C) as previously described. These include:

- Metabolic stimuli: arterial CO_2, extracellular pH, adenosine release, lactate accumulation, elevated extracellular potassium – vasodilators.[11-16]
- Neurogenic stimuli/NVC: synaptic activation, astrocyte calcium signalling – vasoactive.[11, 13, 17]
- Endothelial stimuli: NO – vasodilator, endothelin – vasoconstrictor.[16, 18-21]
- Inflammatory mediators: cytokines with vasoactive properties.[22, 23]

These non-pressure stimuli can directly alter vascular diameter, independently of ABP changes, resulting in concurrent changes in CaBV, ICP, and CBF. When intracranial compliance is high, and ABP and CPP are relatively stable, these effects may not translate into strong observable correlations, and indices such as Mx fluctuate around zero. In contrast, under low compliance, volume-driven changes in ICP can significantly alter CPP in the opposite direction. This inverse relationship between CPP and CBv results in a negative CPP–CBv correlation, reflected by a negative Mx.

Because the same stimulus simultaneously affects CBv and ICP from changes in vascular diameter, the correlations of ABP with CBv (Mxa) and with ICP (PRx) tend to be similar in magnitude and direction. However, these changes occur independently of ABP, meaning that ABP is not the "driver" in this correlation, and true ABP-driven autoregulation cannot be inferred.

Intracranial Baroreceptor Activation and Cushing's Response

The existence of intracranial baroreceptors has been postulated in several studies,[24-26] and was recently demonstrated in experimental work in rats.[27] At very high ICP, further increases in ICP can elicit a compensatory rise in ABP to preserve CPP and CBF, an effect known as the Cushing response (Fig. 3D).[28] Under conditions of elevated ICP and low intracranial compliance, CPP drops well below the lower limits of autoregulation and reactivity, and vessels become passively constricted as the intraluminal pressure decreases.

An increase in ICP may compress the brainstem and activate intracranial baroreceptors, thought to be located in this region. This triggers sympathetic activation and widespread peripheral vasoconstriction, resulting in a rise in ABP, which is reflected by a positive PRx. The relative balance between changes in ABP and ICP then determines the observed patterns in CPP, CBF, and autoregulatory indices:

- If the ABP increase is less than the ICP increase, CPP continues to fall, leading to further passive vasoconstriction, increased vascular resistance, and reduced CBF. This scenario results in a positive Mx and negative Mxa.
- If the ABP and ICP increases are proportional, CPP remains stable, with minimal changes in vessel diameter or CBF, yielding Mx ≈ 0 and Mxa ≈ 0.
- If the ABP increase exceeds the ICP rise, CPP improves, leading to passive vasodilation. In this scenario, CPP remains below the lower limit of reactivity, but the increase in intravascular pressure forces the vessels open, raising CaBV. This results in reduced vascular resistance and a partial or full restoration of CBF, reflected by positive Mx and Mxa.

In the latter case, however, increased CaBV from vasodilation may raise ICP further, initiating a vicious cycle that risks rapid decompensation. Without timely and aggressive intervention, this cascade may progress to brain herniation and ultimately cerebral ischaemia.

C – Translating the Framework: Clinical Utility and Personalised Management

The following abbreviations are used in the upcoming figures: ABP, Arterial blood pressure (mmHg); AMP, Intracranial pressure amplitude; Ca, Vascular compliance; Ci, Intracranial compliance; CrCP, Critical closing pressure (mmHg); CPP, Cerebral perfusion pressure (mmHg); dCM, Diastolic closing margin (mmHg); EtCO2, End-tidal carbon dioxide (mmHg); CBv, Cerebral blood velocity (cm/s); ICP, Intracranial pressure (mmHg); Mx, Mean flow index; Mxa, Arterial mean flow index; PRx, Pressure reactivity index; Ra, Vascular resistance; RAP, Pressure-volume compensatory reserve index; τ, Time constant (s); WT, Wall tension (mmHg); X_f, X signal in the frequency domain (Hz).

1. Patterns of Agreement and Divergence Across Autoregulation Metrics

Case 2: Preserved Autoregulation with Concordant Indices

The visualisation of effective cerebral autoregulation in Case 2 (PRx = -0.6; Mx = Mxa = 0.1; Fig. S1) demonstrates a coordinated myogenic response occurring in the context of preserved autoregulatory function, high intracranial compliance, and a substantial compensatory reserve. This physiological state corresponds to Phase 3 of the autoregulation model (Fig. 2C, left panel). Despite stable $EtCO_2$ and CBv, prominent low-frequency oscillations (≈0.005 Hz) are evident in ABP (ranging from 90 to 100 mmHg) and are reflected in corresponding CPP variations (75 to 88 mmHg). In this setting, vascular compliance fluctuates out of phase with CPP, while vascular resistance and arterial wall tension exhibit a synchronous response, consistent with effective dynamic regulation of vessel calibre.

These phasic changes reflect the ability of cerebral vessels to dilate and constrict in response to fluctuating perfusion pressures, thereby accommodating the surges in blood flow without marked increases in CBv. The resultant modulation of vessel diameter contributes to variations in CaBV, such that a reduction in CPP is met with a compensatory increase in CaBV, indicative of vascular recruitment and reserve capacity. These intracranial volume shifts lead to corresponding oscillations in ICP (12 to 14 mmHg), which closely mirror changes in CaBV. The high intracranial compliance and low RAP observed in this case further support the presence of a robust compensatory reserve, facilitating effective ICP stabilisation in response to transient changes in intracranial volume, whether from cerebral blood, cerebrospinal fluid, or interstitial fluid dynamics.

Together, these findings illustrate a physiologically coherent pattern of intact autoregulatory function across both pressure and flow domains.

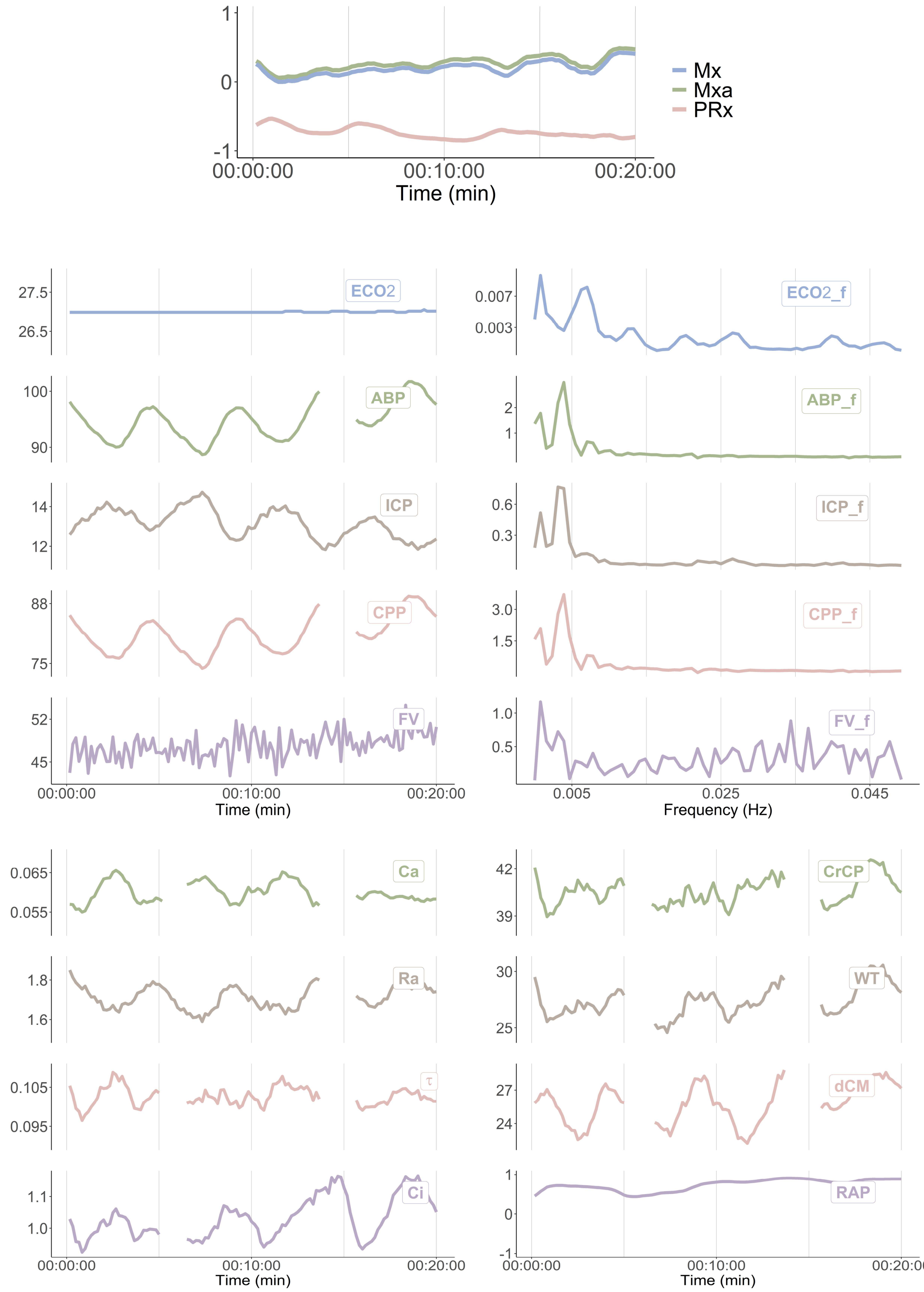

Fig. S1. Case 2, representing Phase 3 (i.e. working cerebrovascular reactivity, working cerebral blood flow autoregulation). EtCO2 and CBv remain constant, while ABP and ICP show long-term variations in opposite directions. Ci is high, with RAP remaining low. Ca, Ra and WT are responsive and indicate reactive myogenic responses.

Case 3: Impaired Autoregulation in the Context of Impending Septic Shock

Case 3 depicts a loss of autoregulatory function across all indices (PRx = 1, Mx = 1, Mxa = 1; Fig. S2), captured during a period preceding septic shock, a condition previously associated with autoregulatory failure.[29, 30] Pronounced low-frequency oscillations (≈0.006 Hz) are observed across ABP, ICP, CPP, and CBv, with $EtCO_2$ remaining stable. During this interval, ABP shows a downward trend, and CPP remains low (<50 mmHg), ultimately falling below 20 mmHg within 30 minutes of the recorded segment. By that time, the diastolic closing margin is reduced to just 5 mmHg.

The negative RAP and diminished arterial wall tension suggest partially collapsing vessels under low pressure. The autoregulatory indices indicate the CPP is well below the lower limit of autoregulation, and the reduced intracranial compliance reflects depletion of intracranial compensatory mechanisms, allowing volume shifts to translate directly into ICP changes. At the vascular level, vascular compliance, vascular resistance, and cerebrovascular time constant all lie at the lower end of their ranges, with patterns consistent with an impaired myogenic response. Interpreting vascular compliance requires recognising its dual dependence on both passive vessel elasticity and active smooth muscle tone. In passive collapse, smaller vessels yield increased vascular resistance and a steeper pressure-volume curve, meaning higher elastance, leading to lower apparent compliance. Conversely, under intact cerebral autoregulation (Phase 3), a fall in CPP leads to vasodilation: vascular resistance decreases, and active muscle relaxation raises vascular compliance.

In this case, cerebrovascular time constant increases as CPP decreases, an indication that passive narrowing predominates, consistent with autoregulatory failure (Fig. 2A, right panel). This contrasts with the autoregulated state of Case 2, where the cerebrovascular time constant is more stable, reflecting effective vascular adaptation. This pattern illustrates how cerebrovascular time constant and its components can help differentiate between passive vascular collapse and active autoregulatory compensation.

The paradoxically elevated CBv observed despite low CPP and impaired cerebral autoregulation may not indicate increased CBF, but rather reflect passive narrowing of the middle cerebral artery. Such constriction would have a minimal effect on total vascular resistance, given the relatively small contribution of the middle cerebral artery to overall vascular resistance. However, it could markedly increase local CBv measurements, as seen in cases of vasospasm. It is also possible that the patient had a

congenitally or anatomically narrow middle cerebral artery segment, which could account for the disproportionately high CBv readings.

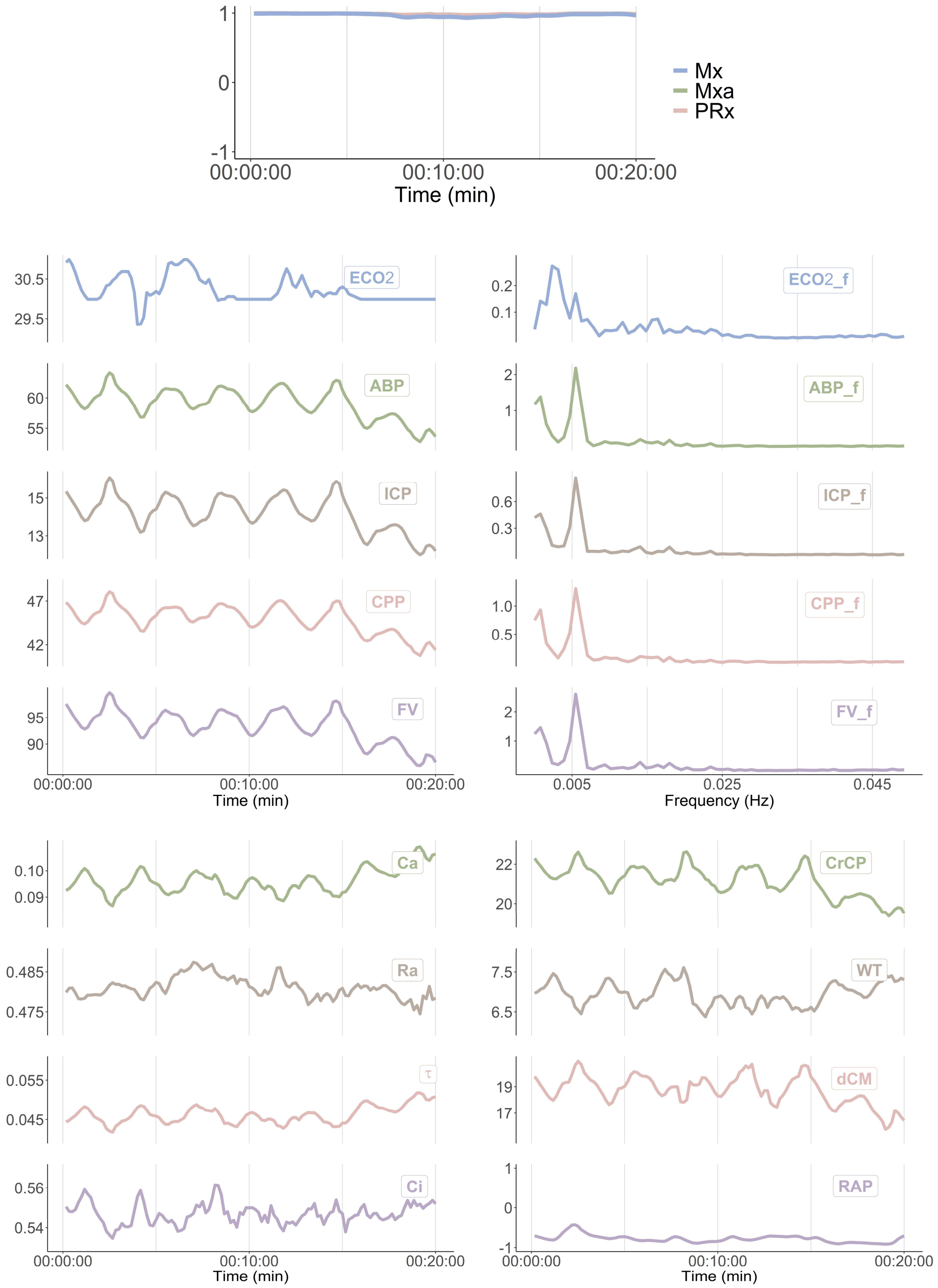

Fig. S2. Case 3, representing Phase 1 (i.e. impaired cerebrovascular reactivity and cerebral blood flow autoregulation). Pronounced oscillations (0.006 Hz) are visible in ABP, ICP, CPP, and CBv, while EtCO2 remains stable. CPP is <50 mmHg. Most physiological parameters (Ca, Ra, WT, Ci) are at the lower end of their ranges, and RAP is particularly negative.

Case 4: Impaired Pressure Reactivity with "Preserved" Flow Regulation – False Autoregulation

Case 4 (Fig. S3) illustrates Phase 6 of the autoregulatory response (Fig. 3B, right panel), characterised by markedly reduced intracranial compliance and depleted compensatory reserve. This is reflected in both low intracranial compliance and high RAP, indicating that even minor changes in CaBV produce proportionate changes in ICP.

Vascular resistance and arterial wall tension are elevated, consistent with pronounced vasoconstriction, while CBv remains low. Notably, vascular resistance varies inversely with CPP, consistent with passive vascular responses to pressure changes. Although ICP remains low, small fluctuations in ABP are efficiently transmitted to ICP (≈0.01 Hz), resulting in a nearly flat CPP trace.

Due to this flat CPP profile, fluctuations in CBv, though present, exhibit weak correlation with CPP, yielding an Mx near zero. In contrast, both PRx and Mxa are elevated (≈0.5), reflecting a positive correlation between ABP and ICP, and between ABP and CBv, respectively.

This phenomenon has been described as false autoregulation in the literature.[31-35] It occurs, as described above, when CPP remains stable because ICP changes counterbalance ABP changes, masking underlying CBF variability, or leading to stabilisation of CBF. False autoregulation is reported in cases of cerebral oedema, haemorrhage, cerebrospinal fluid accumulation, plateau waves, or Cushing response (i.e., ICP elevation). In this context, Mx underestimates the degree of autoregulatory impairment, not due to preserved flow control, but due to the lack of CPP dynamic variability. This case highlights the importance of considering PRx and Mxa alongside Mx, particularly when interpreting flow-based indices under flat CPP conditions.

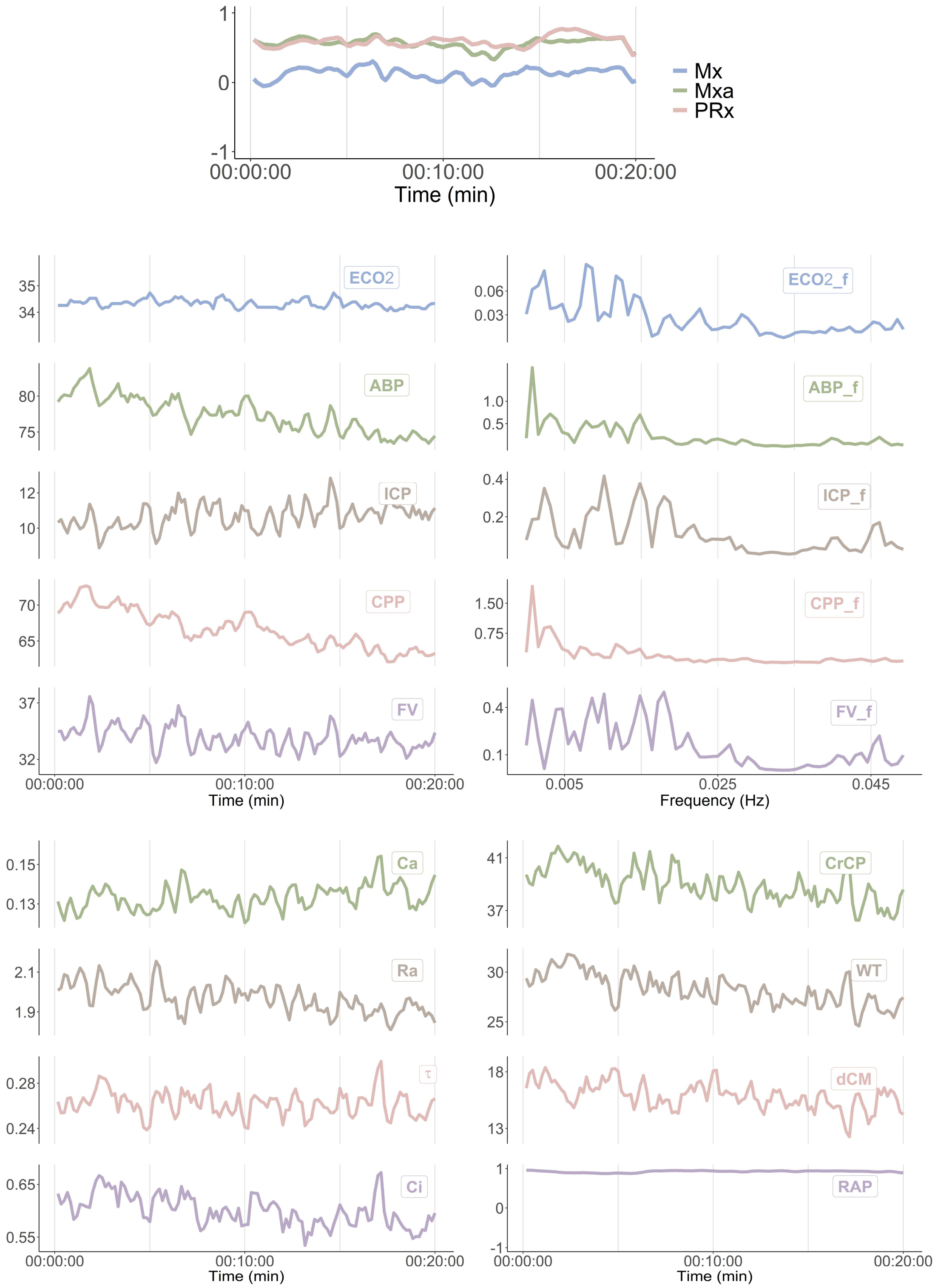

Fig. S3. Case 4, representing Phase 6 (i.e. impaired cerebrovascular reactivity and cerebral blood flow autoregulation) in the case of low Ci, supposedly representing a case of false autoregulation. ABP exhibits some small variations, much more visible in ICP and CBv (0.01 Hz), but absent in CPP. RAP is close to 1, while Ra and WT are in the higher range and not responsive to changes in ABP, as Ca.

2. Violations of Metric Assumptions and Their Consequences

Case 8: Interplay of External Properties and High Intracranial Compliance

PRx relies on the transmission of blood volume changes to ICP. High intracranial compliance can attenuate this transmission, potentially rendering PRx unreliable. This is exemplified in Case 8 (Fig. S4), involving a patient with a progressive tension pneumocephalus.[36, 37] The air accumulation led to a gradual rise in ICP (from 10 to 15 mmHg) and a concurrent increase in intracranial compliance (from 1.5 to 3.5; Fig. S5), while CBv remained low on the ipsilateral side.

This paradoxical elevation in intracranial compliance, alongside a low RAP, likely reflects the altered pressure-volume relationship caused by the physical properties of intracranial air. As a compressible and redistributable medium, air may artificially augment compensatory reserve, flattening the compliance curve.

During a 20-minute window (Fig. S4), ABP, ICP, and CBv remained relatively stable with modest late increases, while $EtCO_2$ was constant. Over this period, PRx, Mx, and Mxa diverged considerably: PRx rose from ≈0 to 0.9, while Mx and Mxa ranged from -0.4 to +0.8. These discrepancies occurred despite minimal slow-wave activity and likely reflect the breakdown of assumptions required for these indices to remain valid.

The findings demonstrate that high compliance, particularly when due to pathologies such as pneumocephalus, can obscure true cerebrovascular reactivity. In such contexts, the reliability of autoregulatory indices must be critically reassessed, especially when transmission of volume change to ICP is non-linear or externally altered.

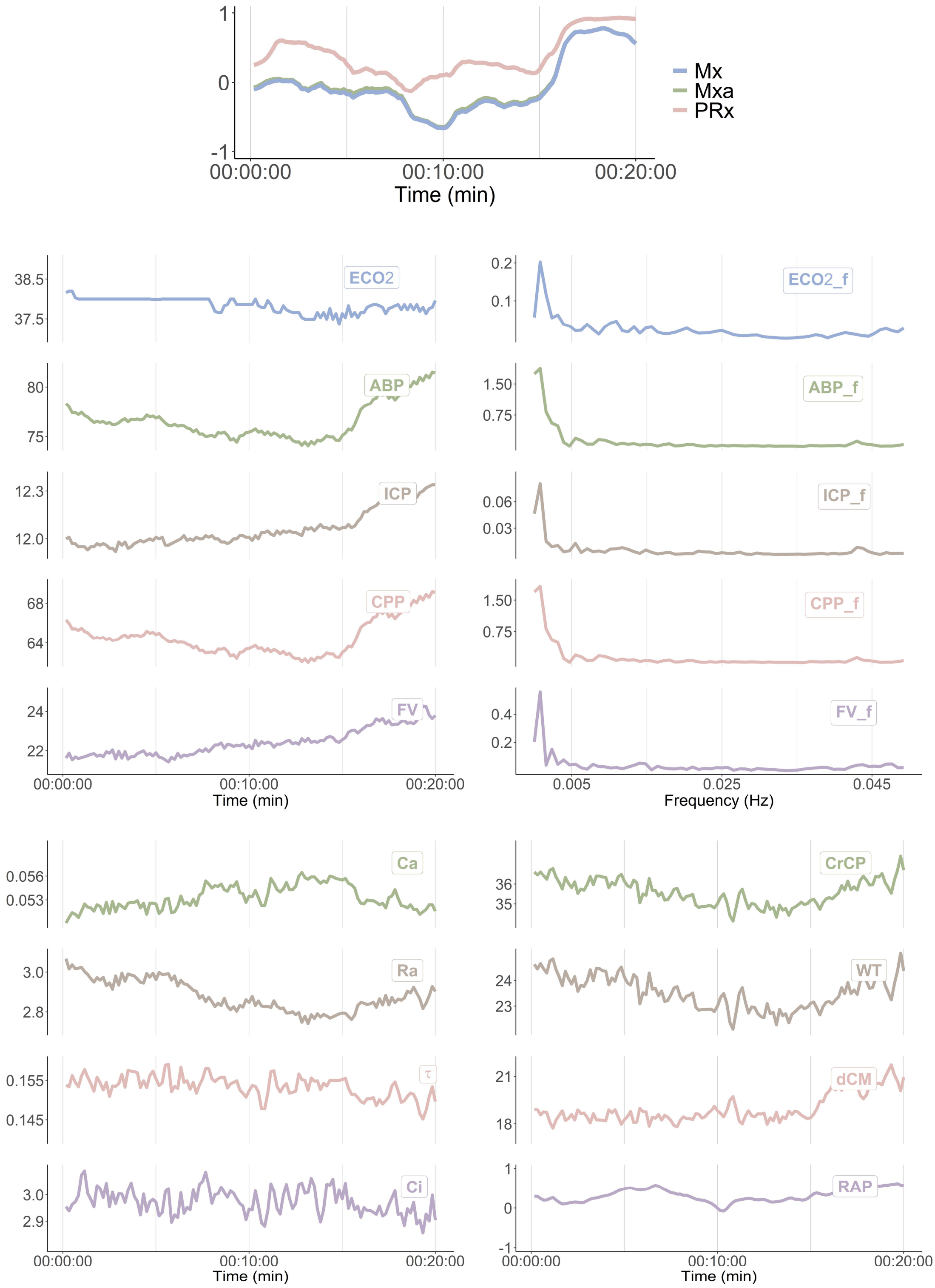

Fig. S4. Case 8 illustrating violation of PRx and Mx assumptions due to tension pneumocephalus. 20-minute section with all physiological parameters showing instability in the conclusions drawn from PRx, Mx and Mxa as three states are visible (PRx > 0, Mx ≈ Mxa ≈ 0; PRx ≈ 0, Mx < 0, Mxa < 0; PRx > 0, Mx > 0, Mxa > 0), with Ci = 3. ABP, ICP and CBv are constant, showing a final rise of varying amplitude, while EtCO2 remains constant. Ca, Ra and WT are relatively constant with small relative changes.

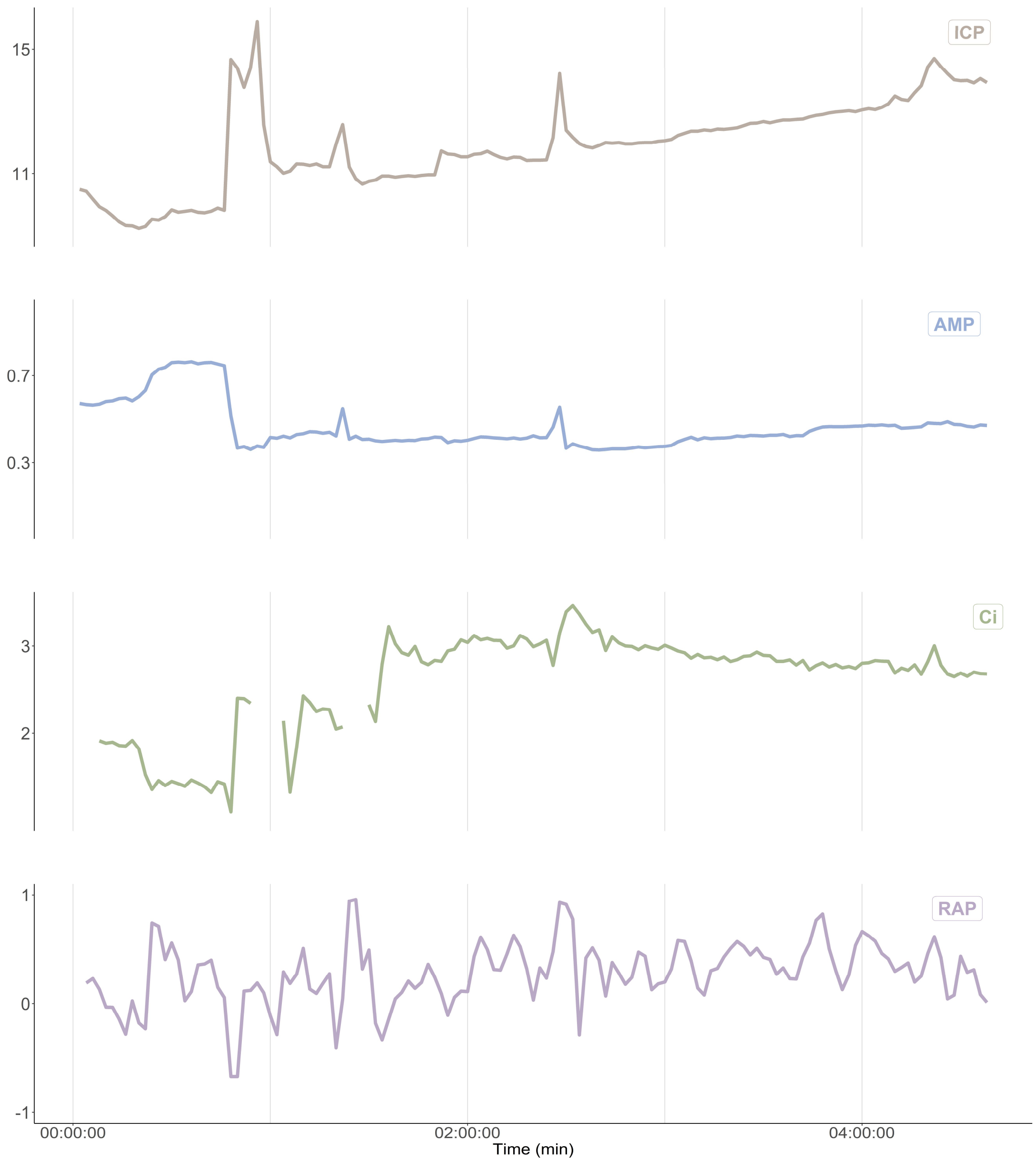

Fig. S5. Case 8 illustrating violation of PRx and Mx assumptions due to tension pneumocephalus. Overall trend of mean ICP, AMP, Ci and RAP. An increase in ICP over the whole recording period is observed from 9 to 15 mmHg, with Ci increasing from 1.5 to 3.5. RAP remains around 0, as AMP is low (0.3) and constant.

Case 9: Reactivity–Autoregulation Dissociation in Extreme Compliance

Case 9 (Fig. S6) features a patient who had undergone primary bilateral decompressive craniectomy and displayed persistently negative PRx values throughout monitoring, potentially reflecting preserved cerebrovascular pressure reactivity. The analysed segment, recorded three days post-decompressive craniectomy, shows a pronounced decline in ABP (from 95 to 70 mmHg), accompanied by increased vascular compliance, reduced vascular resistance, and a lower arterial wall tension, all consistent with vasodilation. Simultaneously, CBv decreased, likely due to excessive vasodilation, reduced CPP, and impaired autoregulatory control of flow. These impairments were captured by Mx and Mxa, both equal to 1. This physiological profile indicates a rise in CaBV, which may contribute to sustained ICP elevation despite decompression. The transmission of ABP fluctuations into ICP was minimal, with only a ≈1 mmHg increase in ICP, despite large ABP variations, attributable to the very high intracranial compliance. Notably, ICP changes occurred in the opposite direction to ABP, resulting in a strongly negative PRx (PRx = -1). Despite this, RAP remained relatively high, suggesting that while compliance was elevated, compensatory reserve was reduced.

This constellation of findings – preserved vessel reactivity (as seen in PRx), yet failure to sustain adequate flow (Mx = Mxa = 1) – reflects a decoupling between pressure and flow regulation (Fig. 2B, left panel). A comparable pattern was observed in Case 1 (Fig. 4), reinforcing the idea that high compliance and autoregulation are not always synonymous with intact perfusion. Crucially, this case underscores that PRx and Mx offer complementary but context-dependent insights. PRx captures an approximate global cerebrovascular reactivity but may be rendered unreliable if increased compliance prevents effective transmission of blood volume changes to ICP, especially post-decompressive craniectomy. Conversely, Mx, while more sensitive to regional flow changes, may be confounded by non-autoregulatory causes of reduced flow, such as vasoplegia or low CPP. Thus, in high-compliance states, neither index alone should be used deterministically.

In summary, elevated compliance does not inherently invalidate PRx or Mx, provided that the necessary transmission of ABP slow waves into ICP and CBv is maintained. When this transmission is disrupted, as seen in Cases 7 (Fig. 7) and 8 (Fig. S5), these indices may become misleading. Therefore, incorporating the clinical context, along with other physiological markers (e.g., intracranial compliance, RAP, vascular resistance), and

waveform analysis enhances the interpretive fidelity of autoregulatory metrics in complex clinical environments, such as the post-decompressive craniectomy state.

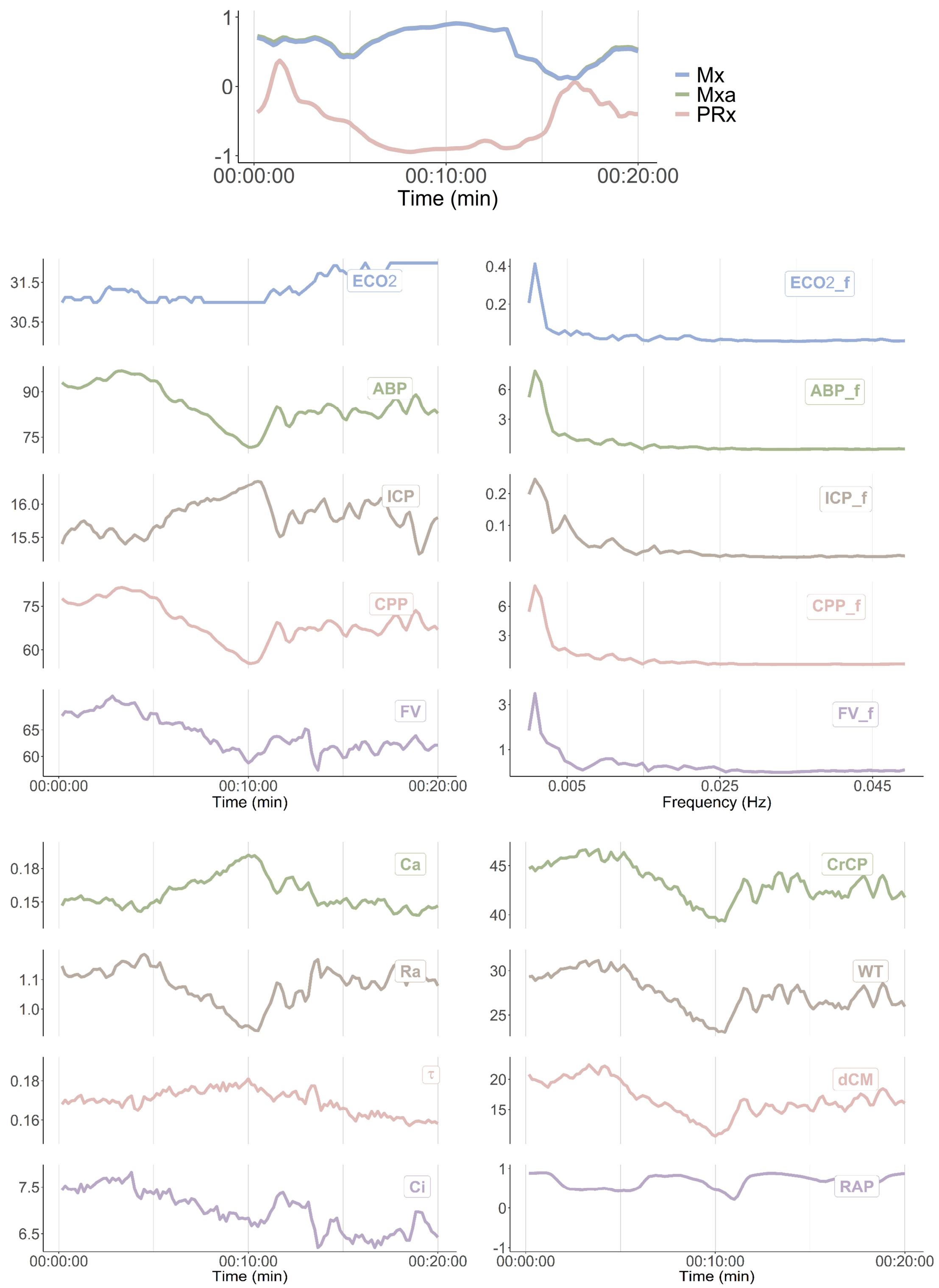

Fig. S6. Case 9, representing Phase 4, with high Ci from decompressive craniectomy. It displays a similar autoregulation state to Case 3, with the particularity of a strikingly high Ci (= 7). A large decrease in ABP (95 to 70 mmHg) is followed by a decline in CBv and a slight increase in ICP of 1 mmHg. Ca increases while Ra and WT decrease significantly. EtCO2 stays constant.

References

1. Bogli SY, Cucciolini G, Cherchi MS, Motroni V, Olakorede I, O'Leary R, et al. Feasibility and Safety of Integrating Extended TCD Assessments in a Full Multimodal Neuromonitoring Protocol After Traumatic Brain Injury. Ultrasound Med Biol. 2024;50(11):1704-15.

2. Smielewski P, Beqiri E, Mataczynski C, Placek M, Kazimierska A, Hutchinson PJ, et al. Advanced neuromonitoring powered by ICM+ and its place in the Brand New AI World, reflections at the 20th anniversary boundary. Brain Spine. 2024;4:102835.

3. Kazimierska A, Kasprowicz M, Czosnyka M, Placek MM, Baledent O, Smielewski P, et al. Compliance of the cerebrospinal space: comparison of three methods. Acta Neurochir (Wien). 2021;163(7):1979-89.

4. Czosnyka M, Smielewski P, Kirkpatrick P, Menon DK, Pickard JD. Monitoring of cerebral autoregulation in head-injured patients. Stroke. 1996;27(10):1829-34.

5. Varsos GV, Richards H, Kasprowicz M, Budohoski KP, Brady KM, Reinhard M, et al. Critical closing pressure determined with a model of cerebrovascular impedance. J Cereb Blood Flow Metab. 2013;33(2):235-43.

6. Kasprowicz M, Diedler J, Reinhard M, Carrera E, Smielewski P, Budohoski KP, et al. Time constant of the cerebral arterial bed. Acta Neurochir Suppl. 2012;114:17-21.

7. Varsos GV, Richards HK, Kasprowicz M, Reinhard M, Smielewski P, Brady KM, et al. Cessation of diastolic cerebral blood flow velocity: the role of critical closing pressure. Neurocrit Care. 2014;20(1):40-8.

8. Czosnyka Z, Lalou A, Pelah AI, Joanides AJ, Smielewski P, Placek MM, et al. Cerebral hemodynamic monitoring combined with infusion test in hydrocephalus. Brain Spine. 2023;3:102705.

9. Bogli SY, Beqiri E, Olakorede I, Cherchi MS, Smith CA, Chen X, et al. Unlocking the potential of high-resolution multimodality neuromonitoring for traumatic brain injury management: lessons and insights from cases, events, and patterns. Crit Care. 2025;29(1):139.

10. Czosnyka M, Guazzo E, Whitehouse M, Smielewski P, Czosnyka Z, Kirkpatrick P, et al. Significance of intracranial pressure waveform analysis after head injury. Acta Neurochir (Wien). 1996;138(5):531-41; discussion 41-2.

11. Gordon GR, Choi HB, Rungta RL, Ellis-Davies GC, MacVicar BA. Brain metabolism dictates the polarity of astrocyte control over arterioles. Nature. 2008;456(7223):745-9.

12. Battisti-Charbonney A, Fisher J, Duffin J. The cerebrovascular response to carbon dioxide in humans. J Physiol. 2011;589(Pt 12):3039-48.

13. Peterson EC, Wang Z, Britz G. Regulation of cerebral blood flow. Int J Vasc Med. 2011;2011(1):823525.

14. Boedtkjer E. Acid-base regulation and sensing: Accelerators and brakes in metabolic regulation of cerebrovascular tone. J Cereb Blood Flow Metab. 2018;38(4):588-602.

15. Duffin J, Mikulis DJ, Fisher JA. Control of Cerebral Blood Flow by Blood Gases. Front Physiol. 2021;12:640075.

16. Mascarenhas A, Braga A, Majernikova SM, Nizari S, Marletta D, Theparambil SM, et al. On the mechanisms of brain blood flow regulation during hypoxia. J Physiol. 2025;603(8):2263-80.

17. Attwell D, Buchan AM, Charpak S, Lauritzen M, Macvicar BA, Newman EA. Glial and neuronal control of brain blood flow. Nature. 2010;468(7321):232-43.

18. Ehrenreich H, Kehrl JH, Anderson RW, Rieckmann P, Vitkovic L, Coligan JE, et al. A vasoactive peptide, endothelin-3, is produced by and specifically binds to primary astrocytes. Brain Res. 1991;538(1):54-8.

19. Macrae IM, Robinson MJ, Graham DI, Reid JL, McCulloch J. Endothelin-1-induced reductions in cerebral blood flow: dose dependency, time course, and neuropathological consequences. J Cereb Blood Flow Metab. 1993;13(2):276-84.

20. Oyama H, Suzuki Y, Satoh S, Kajita Y, Takayasu M, Shibuya M, et al. Role of nitric oxide in the cerebral vasodilatory responses to vasopressin and oxytocin in dogs. J Cereb Blood Flow Metab. 1993;13(2):285-90.

21. Faraci FM. Effects of endothelin and vasopressin on cerebral blood vessels. Am J Physiol. 1989;257(3 Pt 2):H799-803.

22. Ohkawa F, Ikeda U, Kanbe T, Kawasaki K, Shimada K. Effects of inflammatory cytokines on vascular tone. Cardiovasc Res. 1995;30(5):711-5.

23. Iversen PO, Nicolaysen A, Kvernebo K, Benestad HB, Nicolaysen G. Human cytokines modulate arterial vascular tone via endothelial receptors. Pflugers Arch. 1999;439(1-2):93-100.

24. Schmidt EA, Despas F, Pavy-Le Traon A, Czosnyka Z, Pickard JD, Rahmouni K, et al. Intracranial Pressure Is a Determinant of Sympathetic Activity. Front Physiol. 2018;9:11.

25. Marina N, Christie IN, Korsak A, Doronin M, Brazhe A, Hosford PS, et al. Astrocytes monitor cerebral perfusion and control systemic circulation to maintain brain blood flow. Nat Commun. 2020;11(1):131.

26. Vari S, Guild SJ, George B, Ramchandra R. Intracranial baroreflex is attenuated in an ovine model of renovascular hypertension. Sci Rep. 2021;11(1):5816.

27. Wittenberg P, McBryde FD, Korsak A, Rodrigues KL, Paton JFR, Marina N, et al. On the regulation of arterial blood pressure by an intracranial baroreceptor mechanism. J Physiol. 2025;603(9):2517-32.

28. Cushing H. Concerning a definite regulatory mechanism of the vasomotor center which controls blood pressure during cerebral compression. Johnes Hopkins Hosp Bull. 1901;126:290-2.

29. Bindra J, Pham P, Chuan A, Jaeger M, Aneman A. Is impaired cerebrovascular autoregulation associated with outcome in patients admitted to the ICU with early septic shock? Critical Care and Resuscitation. 2016;18(2):95-101.

30. Ferlini L, Su F, Creteur J, Taccone FS, Gaspard N. Cerebral autoregulation and neurovascular coupling are progressively impaired during septic shock: an experimental study. Intensive Care Med Exp. 2020;8(1):44.

31. Miller JD, Garibi J, North JB, Teasdale GM. False Autoregulation after Cold Injury to the Cerebral Cortex. In: Langfitt TW, McHenry LC, Reivich M, Wollman H, editors. Cerebral

Circulation and Metabolism. Berlin, Heidelberg: Springer Berlin Heidelberg; 1975. p. 95-8.

32. Enevoldsen EM, Jensen FT. Autoregulation and CO2 responses of cerebral blood flow in patients with acute severe head injury. J Neurosurg. 1978;48(5):689-703.

33. Sahuquillo J, Amoros S, Santos A, Poca MA, Valenzuela H, Báguena M, et al., editors. False Autoregulation (Pseudoautoregulation) in Patients with Severe Head Injury. Its Importance in CPP Management2000; Vienna: Springer Vienna.

34. Schmidt B, Czosnyka M, Klingelhöfer J. Asymmetry of cerebral autoregulation does not correspond to asymmetry of cerebrovascular pressure reactivity. Perspectives in Medicine. 2012;1(1-12):285-9.

35. van den Dool REC, Sperna Weiland NH, Schenk J, Kho E, Veelo DP, van der Ster BJP, et al. Dynamic cerebral autoregulation during step-wise increases in blood pressure during anaesthesia: A nonrandomised interventional trial. Eur J Anaesthesiol. 2023;40(6):407-17.

36. Harvey JJ, Harvey SC, Belli A. Tension pneumocephalus: the neurosurgical emergency equivalent of tension pneumothorax. BJR case rep. 2016;2(2):20150127.

37. Wankhade BS, Beniamein MMK, Alrais ZF, Mathew JI, Alrais GZ. What should an intensivist know about pneumocephalus and tension pneumocephalus? Acute Crit Care. 2023;38(2):244-8.
